# Kinetics and coexistence of autocatalytic reaction cycles

**DOI:** 10.1038/s41598-024-69267-w

**Published:** 2024-08-08

**Authors:** Balázs Könnyű, Eörs Szathmáry, Tamás Czárán, András Szilágyi

**Affiliations:** 1 Institute of Evolution, HUN-REN Centre for Ecological Research, Konkoly-Thege Miklós út 29-33, Budapest, 1121 Hungary; 2 Center for Conceptual Foundations of Science, Parmenides Foundation, Hindenburgstr. 15., 82343 Pöcking, Germany

**Keywords:** Autocatalysis, Chemostat system, Coexistence, Deep learning, Reaction kinetics, Biochemical reaction networks, Computational models, Machine learning, Biochemical networks, Computer modelling, Differential equations, Dynamic networks, Dynamical systems, Nonlinear dynamics, Numerical simulations

## Abstract

Biological reproduction rests ultimately on chemical autocatalysis. Autocatalytic chemical cycles are thought to have played an important role in the chemical complexification en route to life. There are two, related issues: what chemical transformations allow such cycles to form, and at what speed they are operating. Here we investigate the latter question for solitary as well as competitive autocatalytic cycles in resource-unlimited batch and resource-limited chemostat systems. The speed of growth tends to decrease with the length of a cycle. Reversibility of the reproductive step results in parabolic growth that is conducive to competitive coexistence. Reversibility of resource uptake also slows down growth. Unilateral help by a cycle of its competitor tends to favour the competitor (in effect a parasite on the helper), rendering coexistence unlikely. We also show that deep learning is able to predict the outcome of competition just from the topology and the kinetic rate constants, provided the training set is large enough. These investigations pave the way for studying autocatalytic cycles with more complicated coupling, such as mutual catalysis.

## Introduction

Chemical autocatalysis^1^, like self-replication, is a universal feature of all living systems, also reflected in the fact that it is a regular item on any list of features defining life from a scientific viewpoint^2–5^. Each of the three main functional subsystems of recent living cells: cellular compartment structures (membrane constituents), molecular agents of genetic information transfer (nucleic acids) and the cores of all known metabolic reaction networks, are autocatalytic^6–8^.

According to a feasible prebiotic scenario, the first organic compounds may have originated from reactions of inorganic molecules in the atmosphere of primordial Earth, energized by solar radiation of different wavelengths, and dissolved in the prebiotic oceans^9–16^. There is also ample evidence that organic molecules could have formed in interstellar space, delivered to Earth by meteorites which had intensely bombarded the planet at the time, substantially contributing to the organic content of ancient oceans both in quantity and diversity^17–19^. In the new medium of aqueous solution, new chemical reactions could have taken place among the organic molecules dissolved from the atmosphere and meteorites, or produced by geochemical processes and ejected into the ocean by thermal vents^16,20,21^. With the rich supply of diverse organic compounds and the multitude of different physicochemical environments provided by the ocean and the ocean bed, a vast network of loosely connected organic reactions could have emerged. The pathways of this early reaction network must have been channeled mostly by kinetics lacking catalytic aid, or at best by inorganic catalysts^22–24^. Since these early reaction pathways were not constrained by any functional requirement, the only way they could have produced relatively complex compounds of significance for the earliest stages of chemical evolution towards life was by coincidence, and even so side reactions may have prevailed and turned them into tar^8,9^.

A feasible escape route out of the trap of chemical chaos, even in the absence of specific catalysts, is provided by the kinetic dominance of autocatalytic reaction cycles over linear reaction pathways^25^ or non-autocatalytic cycles. An autocatalytic reaction cycle is a reaction path that forms a closed loop and includes a reaction producing an extra molecule of one of the loop members, A, while the cycle turns around once. From the viewpoint of this molecule, the self-propagating reaction A → 2A takes place in a single turn of the cycle, under the proper conditions and with all necessary resource compounds for all the reactions of the cycle present. Then the amount of molecule A, and, consequently, of all the other cycle members increase exponentially, which can radically change the dynamics of the network of reactions embedding the autocatalytic cycle: its compounds can persist and dominate in spite of the strong chemical noise in non-catalytic chemical networks. The autocatalytic subsystem increases much faster than it is eroded by side reactions. Such a cycle may also become a source of 'feedstock' for the larger network that includes it, as are the biochemical cycles in the cells of all recent organisms: some elements of the Krebs or TCA cycle provide substrates to chemical pathways producing other biomolecules (e.g., amino acids). For each of the best-known autocatalytic cycles with a supposed role in prebiotic (chemical) evolution: the formose reaction^26–29^, the reverse tricarboxylic acid cycle^30^, and the glyoxylate cycle^31,32^, it is also true that individual cycle members can plug into a putative pathway for the production of a biomolecule. Thus, autocatalytic cycles may have emerged from the prebiotic chemical mayhem due to their kinetic advantage, and, once in place, they could have provided a continuous supply of biomolecules for the evolution of higher levels of (pre-)biotic organization. Both of these advantages can be attributed to the potential of autocatalytic cycles to grow exponentially.

**Figure 1:**
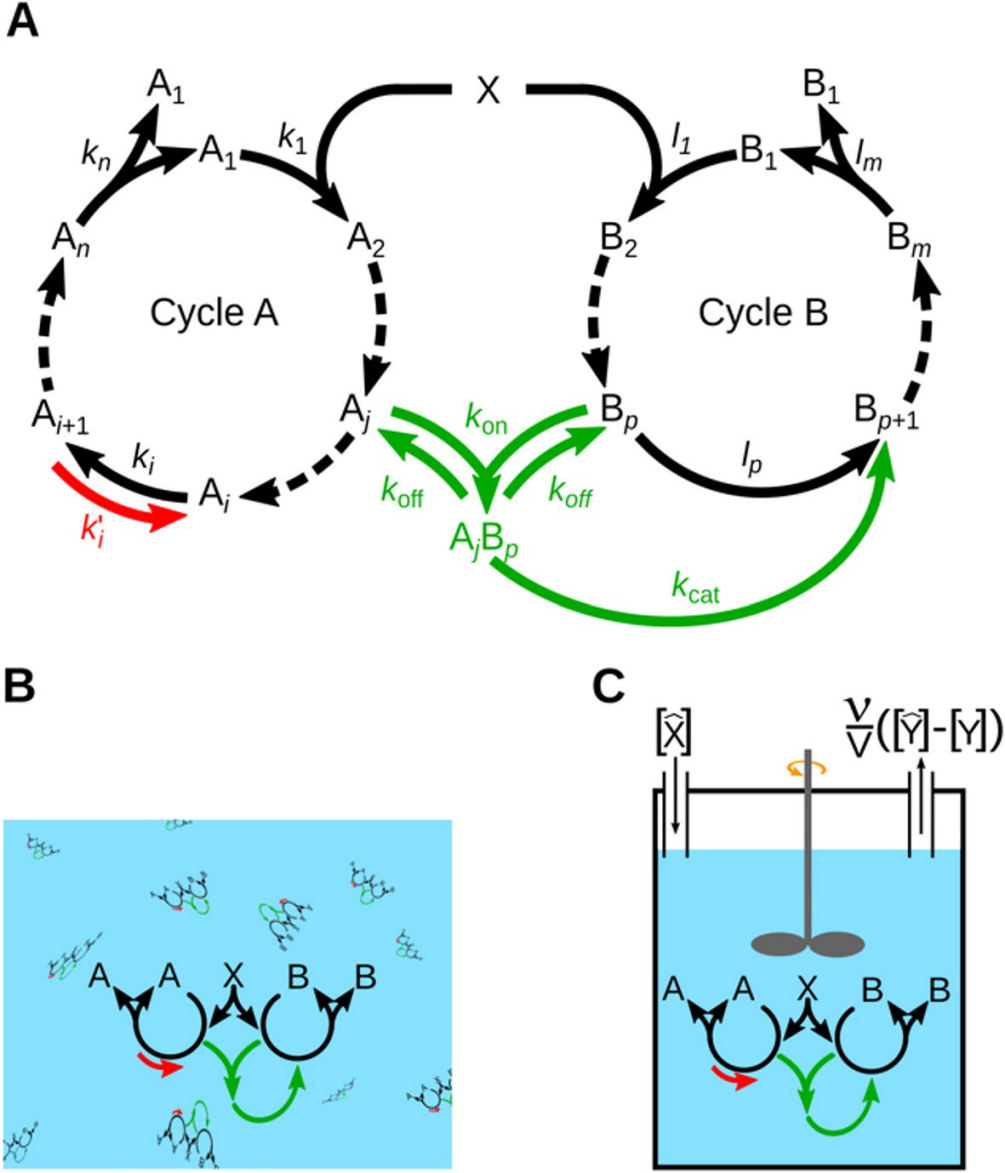
Schematic representations of the competitive autocatalytic cycles in different scenarios. Panel (**A**) Schematic representation of the three main types of reaction involved in our analysis. Black arrows indicate the core reaction sets of cycles, the red arrow indicates a (generalized) reverse reaction in cycle $\text{A}$ (the corresponding rate denoted by a prime), and green arrows represent a monomolecular catalytic reaction scheme (details in the text) between the two cycles ($p>1$). Dashed arrows denote further irreversible core reactions (kinetic constants not shown); Panel (**B**) Caricature of the resource-unlimited system. Panel (**C**) Caricature of the resource-limited (chemostat) system, where $\text{Y}$ stands for any substance present in the chemostat (cycle members and the resource ($\text{Y}\in \left\{{\text{A}}_{1},\dots , {\text{A}}_{n}, {\text{B}}_{1}, \dots , {\text{B}}_{m},\text{ X}\right\}$).

In this study, we have systematically analysed the kinetic feasibility of the persistence of solitary autocatalytic cycles and the coexistence of two concurrent cycles under different resource regimes (unlimited resource supply and constant resource flux into a chemostat), with or without allowing reversible reactions along the cycles, and with or without cross-catalytic interactions between concurrent cycles (Fig. 1). The different scenarios of the simulation studies are detailed in the Methods section.

## Results

### Single autocatalytic cycle in a resource-unlimited system

We analyse four different types of dynamics for a single autocatalytic cycle with unlimited resource replenishment as cases of reference, assuming that the resource is always present in a fixed, unit concentration: $\frac{{\text{d}}\left[\text{X}\right]}{{\text{d}}t}=0, \text{and} \left[\text{X}\right]=1$, and the initial concentrations of all members of the cycles are 0.01 in all simulations. For the details of the analyses, see (Table 1) and Supplementary Information 2 for details.

**Table 1:** The asymptotic behaviour of a single, resource-unlimited autocatalytic cycle under different reversibility assumptions.

Cases	Irreversible	Reversible	Asymptotic behaviour	Figure 2A colour code
(1)	all	none	Exponential growth ($\it {\uplambda }_{1}, {\uplambda }_{1\text{s}}$) Supplementary equation (S9)	Black, red
(2)	last + others (not all)	any but last	Exponential growth ($\it {{\uplambda }_{2}<\uplambda }_{1}$) Supplementary equation (S12)	Dashed green
(3)	any but last	last + others (not all)	Subexponential growth Supplementary equation (S13–S15)	Light green
(4)	none	all	Constant equilibrium Supplementary equation (S16–S18)	Blue

**Figure 2:**
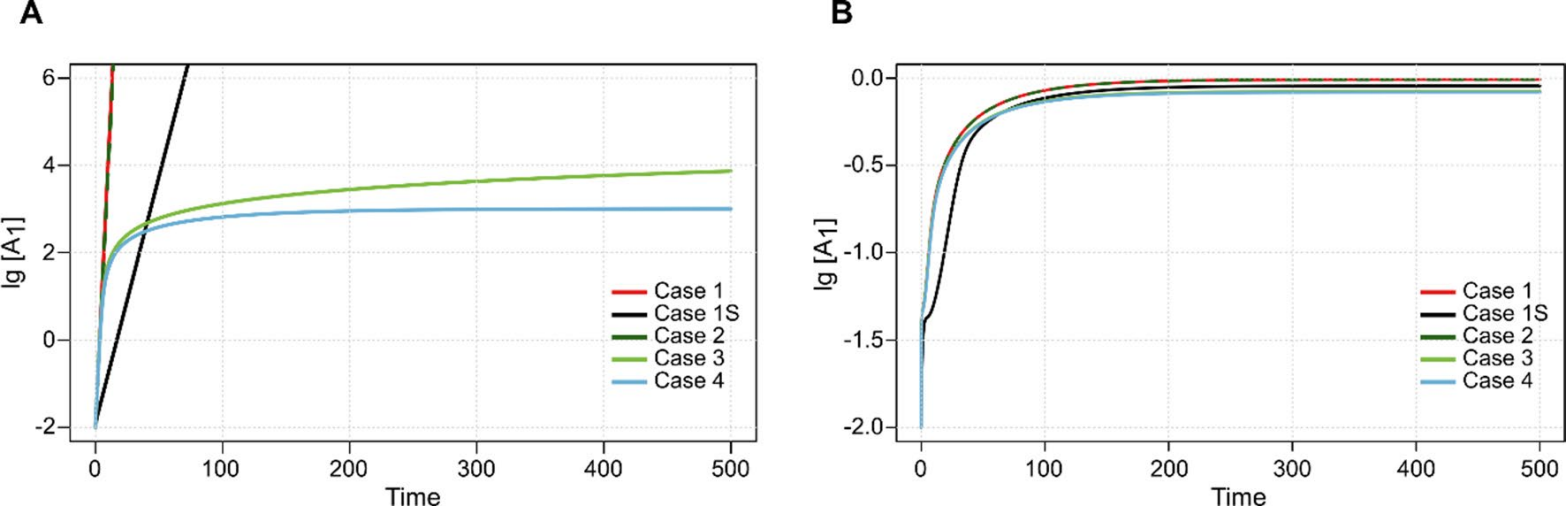
Time courses of the concentration of the first element ($[{\text{A}}_{1}]$) of the autocatalytic cycle in the four scenarios discussed. Panel **A**. Dynamics of resource-unlimited systems. Red curve (Case 1): no reverse reaction (${k}_{1}=5.87, {k}_{2}=3.84, {k}_{3}=7.56$), ${\lambda }_{1}=0.606$; black curve (Case 1S): no reverse reactions, the rate constants are identical (${k}_{1}=1.00, {k}_{2}=1.00, {k}_{3}=1.00$), ${\lambda }_{1\text{s}}=0.113$; dashed green curve (Case 2): the first reaction step is reversible ($k^\prime_1=0.59$, all other rates are as before), ${\lambda }_{2}=0.586$; light green curve (Case 3): the last (doubling) reaction step is reversible ($k^\prime_3=0.76$, all other rates are as in Case 1); blue curve (Case 4): all reactions are reversible ($k^\prime_1=0.59, k^\prime_2=0.38, k^\prime_3=0.76,$ all other rates are as in Case 1). Note the logarithmic scale of the vertical axis. Panel **B**. Chemostat systems. The rate constants and colors are the same as in panel A. In the resource-unlimited simulations the concentration of the resource was fixed at $\left[\text{X}\right]=1.0$; the initial concentrations in all simulations were $\left[{\text{A}}_{1}\right]\left(0\right)=\left[{\text{A}}_{2}\right]\left(0\right)=\left[{\text{A}}_{3}\right]\left(0\right)=0.01.$

*Case 1 (fully irreversible cycle).* If all forward kinetic rate constants are positive, and no reverse reactions are allowed, the system grows exponentially with growth rate ${\lambda }_{1}$ (Fig. 2A, red curve). The implicit equation defining this growth rate is the following:

1 $$\mathop \prod \limits_{i = 1}^{n} \left( {\lambda_{1} + k_{i} } \right) = 2\mathop \prod \limits_{i = 1}^{n} k_{i} ,$$

which has a single positive root: the growth rate of the system (cf. Supplementary equation (S9)). In general, there is no closed analytical form can be computed for ${\lambda }_{1}$ except if all forward rate constants are equal ($k={k}_{i}$), in which case the growth rate $({\lambda }_{1s}$) is (see Fig. 2A, black curve; cf. Supplementary equation (S11):

2 $$\lambda_{{1{\text{s}}}} = \left( {\sqrt[n]{2} - 1} \right)k.$$

*Case 2 (reversible reaction(s) on any internal step, irreversible doubling step).* If any, except the doubling, step can be reversible ($\sum_{i=1}^{n-1}k^\prime_i>0$ and $k^\prime_n=0$) the exponential growth tendency remains but, with a slightly reduced growth rate ${\lambda }_{2}$ compared to ${\lambda }_{1}$ (Table 1, Fig. 2A, dashed green curve; cf. Supplementary equation (S12)). According to our numerical experiences, introducing reversibility on internal step(s) has a very little effect on the growth rate (cf. red and dashed green curve in Fig. 2A).

*Case 3 (cycle not fully reversible, reversible doubling step).* If the last reaction step (the one doubling A_1_) is reversible ($k^\prime_n>0)$, and at least one intermediate reaction is irreversible ($\prod_{i=1}^{n-1}k^\prime_i=0)$ the dynamics become qualitatively different: the overall growth of the cycle follows a subexponential trajectory that seems to be a power (quadratic) function of time (Table 1, Fig. 2A, light green curve and Supplementary Figure S1; cf. Supplementary equation (S13)).

*Case 4 (fully reversible cycle).* Another qualitative change in the dynamics of the system occurs if all the reactions of the cycle (including the doubling step) are reversible ($k^\prime_i>0, i=1,\dots ,n)$. Under this assumption, the system becomes self-regulated in spite of the lack of resource limitation. The overall concentration of the cycle members converges to a fixed point at which the cycle turns around at the same speed in the forward and reverse directions. In this case, the equilibrium concentrations can be computed analytically, see Table 1 and Fig. 2A, blue curve; cf. Supplementary equation (S14-S15).

### Single autocatalytic cycle in a chemostat

The chemostat setting makes the dynamics of a single autocatalytic cycle resource-limited^33^. There are no simple analytical formulae for the equilibrium concentrations, not even in the simplest case; the time course of concentrations can be obtained by numerical integration of (Supplementary equations S3). Figure 2B shows the concentration of ${A}_{1}$ in the chemostat for the four different cases (cf. Cases 1–4 above) corresponding to those in (Fig. 2A). Due to resource limitation arising from the finite replenishment rate of $\text{X}$ into the chemostat through the inlet, and the concentration-dependent harvesting of the system components through the outlet, all concentrations settle on steady-state values in all scenarios^33^.

### Two autocatalytic cycles in a resource-unlimited system

Assuming an unlimited resource supply (i.e., constant $[\text{X}]$) decouples the dynamics of autocatalytic cycles: each one behaves as if the other would not be present. Therefore, in the case of exponentially growing cycles, one excludes (*dilutes*) the other, except for the degenerate (practically implausible) case of exactly equal exponential growth rates (*Case 1*). If any one of the two cycles has at least one irreversible reaction step while the other cycle is fully reversible (i.e., $\prod_{i=1}^{n}k^\prime_i=0$ and $\prod_{j=1}^{m}l^\prime_j>0$ or vice versa, where *n* and *m* are the lengths of the first and the second cycle, respectively), the exponentially (*Cases 1–2*) or subexponentially (*Case 3*) growing irreversible cycle will dilute the fully reversible (*Case 4*) one out: coexistence is not possible. A possible case of coexistence is that of two subexponentially growing cycles (*Case 3*): since the pooled concentration of the constituents of any subexponential cycle appears to increase quadratically with time in the limit, the concentration ratio of two such subexponential cycles remains constant, i.e., neither dilutes the other out, while the growth of both cycles remains unlimited. Stable coexistence of two (or more) cycles requires that both cycles admit a finite fixed point, regardless of the presence of the other. As we have seen earlier, this holds only if the reverse kinetic constants are all positive for both cycles (*Case 4*); in this case, static coexistence is possible.

### Two autocatalytic cycles in a chemostat

To analyze the dynamical behavior of two autocatalytic cycles in a chemostat environment, we assume three types of potential regulating mechanisms between them: competition due to resource limitation, which is constitutive in chemostat systems, and two optional ones: self-regulation through reverse reactions and cross-catalytic activity between the competing cycles (cases S, SX (substrate then resource), XS (resource then substrate), see details in Supplementary Information 1). We will discuss each of these regulatory mechanisms in turn.

#### Fully irreversible autocatalytic cycles

As shown previously, unlike in the resource-unlimited system, the concentrations of the elements of a single autocatalytic cycle always converge to steady-state values in a chemostat. However, ‘competition’ between two fully irreversible autocatalytic cycles ($\sum_{i=1}^{n}k^\prime_i= \sum_{j=1}^{m}l^\prime_j=0$) for the common resource $\text{X}$ (which is supplied at a finite, constant rate into the chemostat) results in one cycle excluding (diluting out) the other. In the following, we will refer to the cycle that excludes the other one in the fully irreversible system in the chemostat regime as the *dominant cycle*. To analyze this setup in detail, we recorded the outcomes of ${10}^{5}$ independent runs with randomly assembled {$k, l$}-sets, using the Monte-Carlo method as described in Supplementary Information 3. If the two cycles were of equal length ($n=m=3$), the simulations yielded competitive exclusion of one or the other cycle with an equal chance for cycle $\text{A}$ and $\text{B}$ to be the winner (see Supplementary Figure S2A, bar ‘None’). That is, the extensive Monte-Carlo analysis convincingly suggests that two irreversible autocatalytic cycles with the same length cannot coexist on a single shared resource.

**Figure 3:**
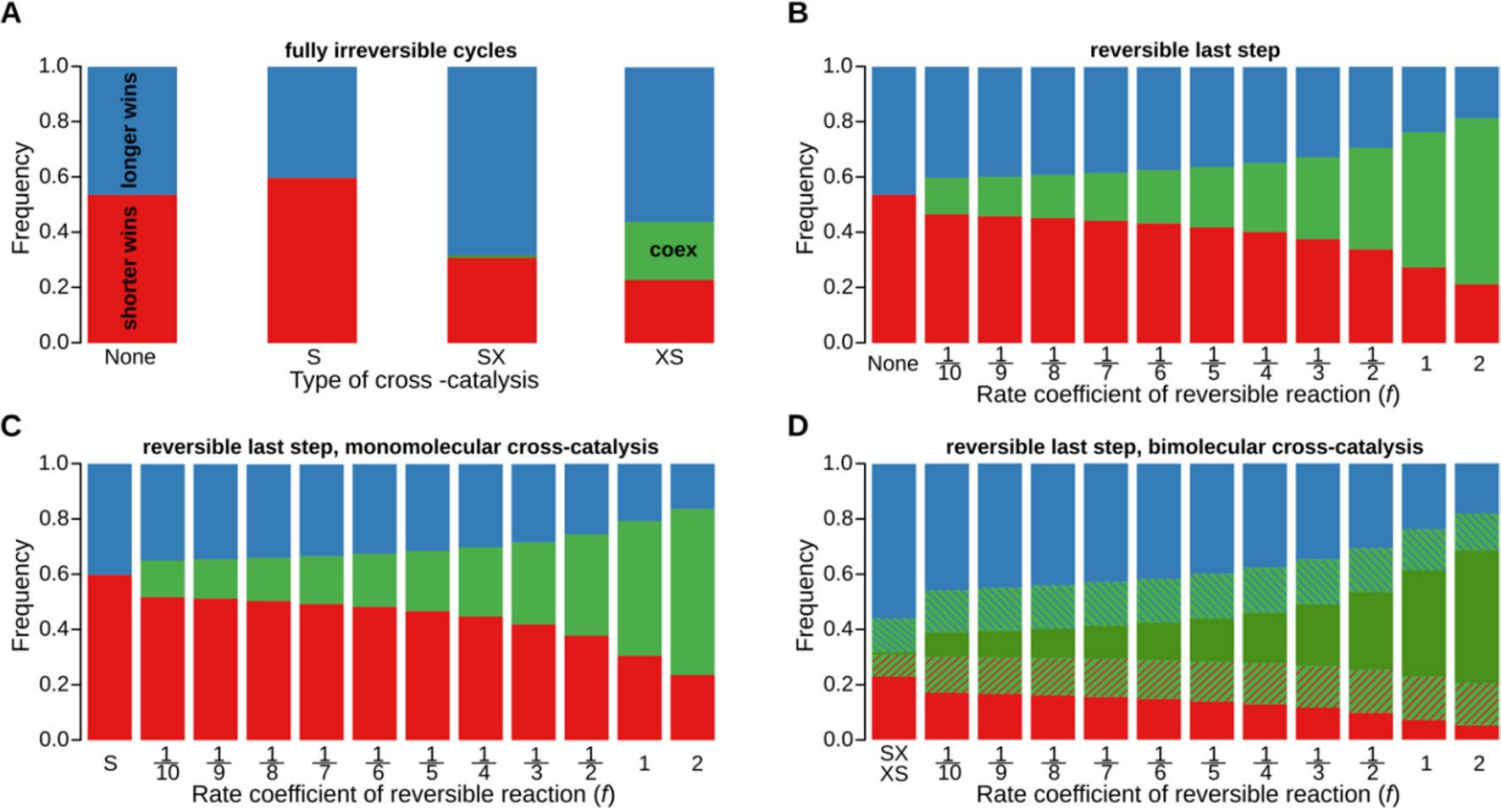
Coexistence and exclusion between two cycles of different lengths in a chemostat. Cycle $\text{A}$ has 3 members ($n=3$), and cycle $\text{B}$ has 7 members ($m=7$). Red: shorter cycle wins; blue: longer cycle wins; green: the two cycles coexist. Panel (**A**) Frequencies of different outcomes of the simulations in case of fully irreversible cycles. Bar ‘None’: no cross-catalysis; bar ‘S’: monomolecular cross-catalysis; bar ‘SX’: bimolecular ‘substrate than resource’ cross-catalysis; bar ‘XS’: bimolecular ‘resource than substrate’ cross-catalysis based. Panel (**B**) The effect of the rate coefficient ($f$) of the reverse reaction on the last step of the dominant cycle. Panel (**C**) The joint effect of a reverse reaction on the last step of the dominant cycle and monomolecular cross-catalysis. Panel (**D**) The effect of a reverse reaction on the last step of the dominant cycle and both types of bimolecular cross-catalytic interaction (SX and XS). Solid and crosshatched red and blue boxes represent the proportion of cases (i.e., {*k,l*}-sets) in which the shorter cycle and the longer cycle wins in the SX scenario, respectively. Dark green boxes show the proportions of the coexistent cases in the SX scenario. Full blue, red and green (light green behind the crosshatched and dark green boxes together) represent the proportion of wins for the shorter cycle, for the longer cycle, and the coexistence of the two cycles, in the XS scenario, respectively. In all scenarios we ran ${10}^{5}$ independent Monte Carlo simulations with random {$k, l$}-sets. Initial concentrations: ${[\text{A}}_{i}]{=[\text{B}}_{j}]=0.01$ for all $i$ and $j$, the resource supply is $\left[\hat{\text{X}}\right]=1.0$, $\nu =0.02, V=1.0$.

Longer cycles turn around more slowly on average, which is reflected in their lower chance of winning (Fig. 3A, bar ‘None’), but the outcome of competition between two irreversible autocatalytic cycles feeding on the same resource of limited supply seems always to be the exclusion of one, irrespective of their lengths). The difference between longer (cycle B, blue) and shorter (cycle A, red) cycles is in their effective growth rates: with more *l* values drawn at random from the same pool for the longer cycle, the effective growth rate it admits can be expected to decrease with system size, in a manner similar to that suggested by Supplementary equation (S11). Obviously, the actual $l$-sets may deviate from the expectation to a large extent, allowing the longer cycle to win in a proportion of cases decreasing with the size difference $\left|n-m\right|$. Generalizing these results, we can say that fully irreversible, resource-limited autocatalytic cycles cannot coexist: the cycle with a higher effective growth rate outcompetes the other. Note that this dilution effect also occurs if more than two cycles depend on the same resource, with a single winner remaining in any such case.

Next, we study the potential regulating effects of introducing the two different optional structural changes in this basic chemostat system: *i)* reverse reactions, and *ii)* cross-catalysis between the cycles (see details in Supplementary Information 3).

#### Reverse reactions

As shown above, allowing reversible reactions in a cycle may qualitatively change the dynamics, the effect depending on the actual positions of the reversible steps. In the reference case without reverse reactions, the simulations with random parameters resulted in the exclusion of cycle $\text{A}$ or $\text{B}$, as explained in the previous section.

**Figure 4:**
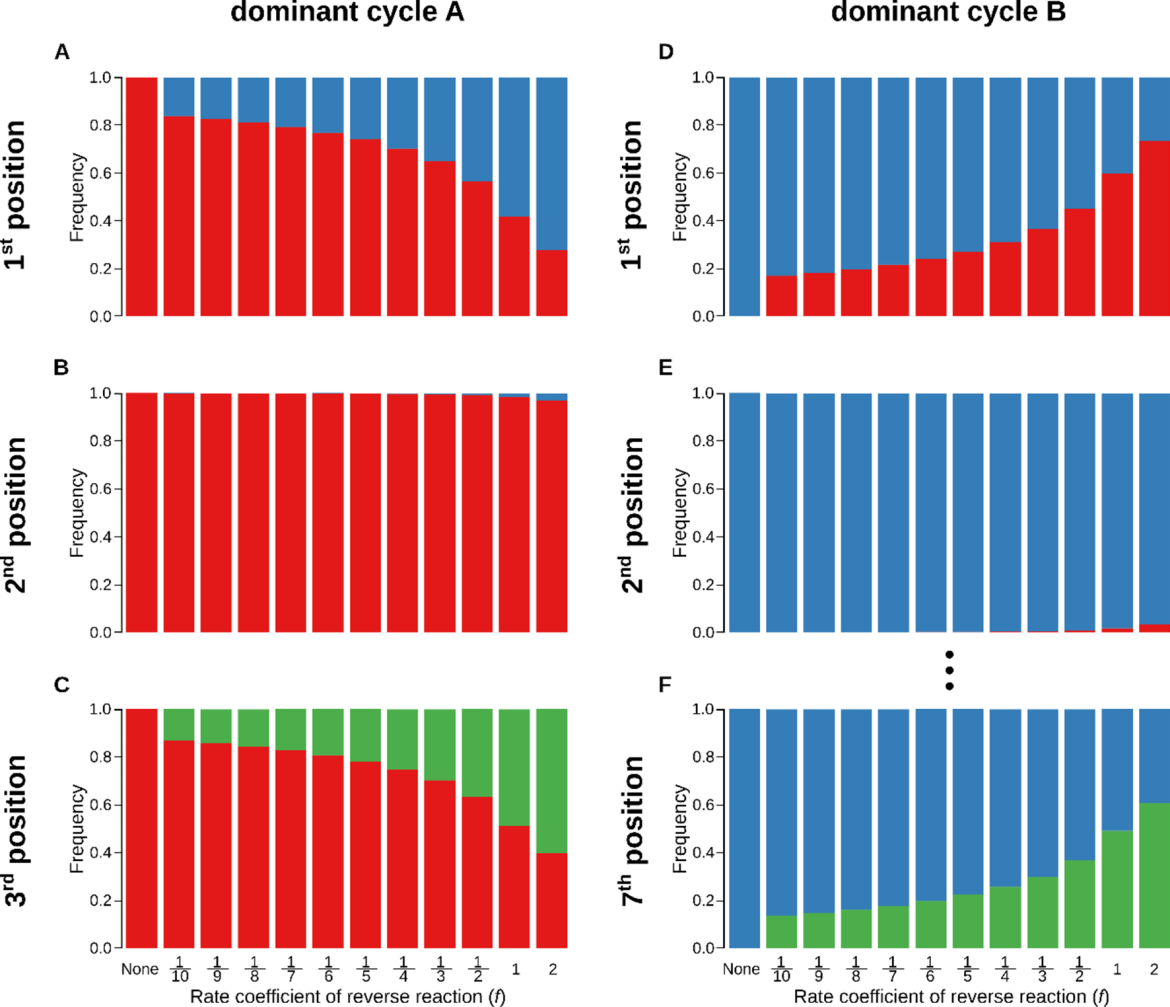
Effect of the position and the reverse rate coefficient ($f$) of a single reverse reaction in the dominant cycle. Cycle $\text{A}$ has 3 members ($n=3$), and cycle $\text{B}$ has 7 members ($m=7$). Left panels (**A**–**C**) show the effect of the reverse reaction at the 1st, 2nd and 3rd reaction step, respectively, when the dominant cycle is $\text{A}$. Right panels (**D**–**F**) show the effect of the reverse reaction at 1st, 2nd and 7th reaction step, respectively, when the dominant cycle is $\text{B}$. In each panel, the reverse rate coefficient increases from left to right. Red: cycle $\text{A}$ wins, blue: cycle $\text{B}$ wins, and green: cycles $\text{A}$ and $\text{B}$ coexist. For each scenario ${10}^{5}$ independent simulations were run with random $\left\{k,l\right\}$ parameter sets. Initial concentrations: ${[\text{A}}_{i}]={[\text{B}}_{j}]=0.01$ for all $i$ and $j$, the resource supply is $\left[\hat{\text{X}}\right]=1.0$, $\nu =0.02, V=1.$

Obviously, any reverse reaction comprises a growth handicap for the cycle whose effective growth rate it decreases, due to the reduced net forward rate of the reaction affected. Our focus of interest is now on the effect of reverse reactions on the coexistence of competing cycles, which clearly cannot occur with the losing cycle depressed even more by a reverse reaction. Therefore, in order to map the effects of reverse reactions on the coexistence of competing cycles in the chemostat, a single random reaction step $i$ from the *dominant* cycle of each simulation was chosen, and its reverse reaction rate $k^\prime_i$' or $l^\prime_i$ (depending on which of the cycles was dominant) was set to the fixed proportion $f$ of the corresponding forward reaction rate: $k^\prime_i=fk_i$ or $l^\prime_i=fl_i.$ Multiple reversible reactions in the dominant cycle were excluded due to the combinatorial explosion of the number of such cases. The following reverse rate coefficients were used in turn for each $\{k,l\}$-set: $k^\prime_i=fk_i$ or $l^\prime_i=fl_i.$ Figure 3B show the frequencies of different outcomes as a function of reverse rate coefficient $f$ in the only case allowing for coexistence, with the reversible reaction at the last step of the dominant cycle (3^rd^ or 7^th^ reaction step, in case of cycle $\text{A}$ and $\text{B}$B as the dominant, respectively) for the different length($n=3, m=7$). The same plot for cycles of equal length ($n=m=3$) is Supplementary Figure S2B. Figure 4 and Supplementary Figure S3 show the effect of the position of the reversible step on the outcome for different and equal cycle sizes, respectively. (Note that Fig. 4 omits the plots corresponding to internal positions 3 to 6 for clarity, these plots can be found in Supplementary Figure S4.)

Any substantial dynamical effect occurs only with the reverse reaction at the first or the last reaction of the dominant cycle. The reverse reaction at the first (resource uptake) step pumps the resource ($\text{X}$) already taken by the dominant cycle back into the chemostat vessel, and the inferior cycle may use it to revert the outcome (i.e., the inferior may exclude the dominant cycle). The probability that such an exclusion swap occurs increases with the speed of the reverse reaction at the resource uptake position, but it cannot maintain the coexistence of the two cycles. Allowing the internal step(s) to be reversible is much less effective (see Fig. 4, Supplementary Figures S4, S3); the effect is also a swap of exclusion at best. On the other hand, allowing the reverse reaction to occur on the last (doubling) step of the dominant cycle can change competitive exclusion to coexistence. This may be related to the fact that making the doubling reaction reversible changes the growth of the autocatalytic cycle from exponential to subexponential (apparently: quadratic in time) even with unlimited resource supply. Positioning the reverse reaction at any of the internal (not the first and not the last) reactions does not substantially influence the dynamics of the system; only very fast reverse reactions in internal positions could flip over the original dominance relation of the autocatalytic cycles. That is, the dynamical effect of reversible internal reaction steps is similar to that of the reverse resource-uptake step, but it is much weaker.

#### Cross-catalysis

The effects of cross catalysis were investigated for the three different scenarios: ‘S’: monomolecular cross-catalysis (Supplementary equation S4a); ‘SX’: bimolecular ‘substrate than resource’ cross-catalysis (Supplementary equation S4b); ‘XS’: bimolecular ‘resource than substrate’ cross-catalysis (Supplementary equation S4c). In case of fully irreversible cycles only XS-type catalysis can promote coexistence at a sizeable part of the parameter space, the other two have marginal effects, see Fig. 3A and Supplementary Figure S2A, bars S, SX and XS. If the last reaction step is reversible S-type catalysis does not substantially change coexistence in any of the simulations (compare Fig. 3B,C and S2B,C), whereas $\text{SX}$ seems to weaken, and $\text{XS}$ to strengthen the propensity for coexistence (Fig. 3D and Supplementary S2D), if the last reactions of the cycles are reversible.

### Patterns of kinetic rates and dynamical classification of competing cycles by deep learning

**Figure 5:**
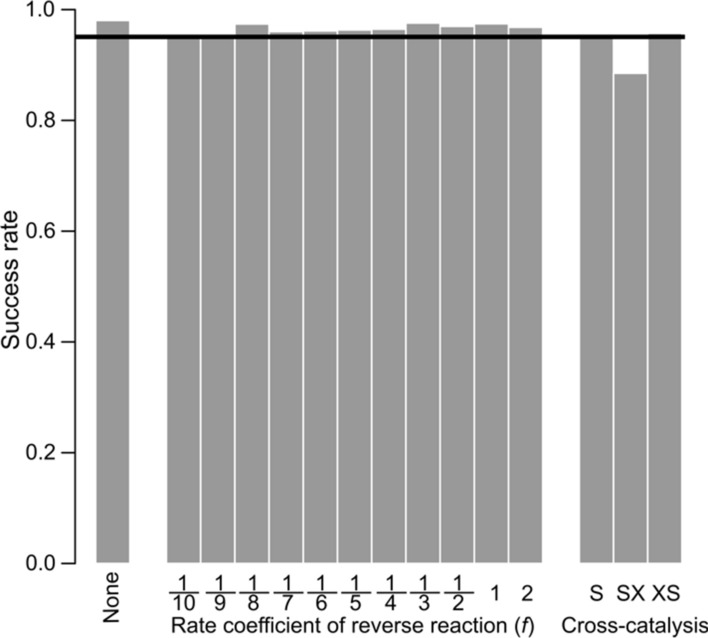
The overall success rates of neural networks. The black line shows the 95% success rate. The sizes of the cycles were the same: $n=m=3$.

The neural network performs a typical classification task: it identifies the outcomes of the numerical integrations based on the patterns of the corresponding kinetic rate values. To validate the reliability of the identification we used independent test datasets sampled from 10 000 numerically integrated $\{k,l\}$-sets for each scenario, for technical details see Supplementary Information 4 and Table S1. The overall success rates (which includes A-win, B-win and coexistence) exceed 95% for almost all scenarios, see in Fig. 5, black line corresponds to the 95%. The results of the detailed analysis can be seen in Supplementary Figure S5. For the *fully irreversible autocatalytic cycles* the trained neural network classifies the outcomes (cycle A wins, or cycle B wins in Supplementary Figures S5B and C bar ‘None’, respectively), the overall success rate is very high (Fig. 5). For the *cycles with a reverse reaction* prediction reliability is also quite high, but it varies irregularly with the rate coefficient of the reverse reaction $f$, see Figs. 5 and S5. Stochastic variation in the success rates is an inherent feature of the deep-learning algorithm: the training set (see Supplementary Table S1) was randomly split into a smaller (validation) and a bigger (training) sub-set during training, and the success of learning depends on the actual composition of these sub-sets. Obviously, increasing the size of the training set decreases the fluctuation in success rate. For the same reason, this stochastic fluctuation in success rates is even more pronounced for the cross-catalysis scenarios due to their extremely small training and test datasets (exactly 17 and 222 coexisting outcomes in 10 000 simulations for S- and SX-type scenarios, respectively, Supplementary Figure S5 and Table S1). We have attempted to explain the outcomes by simple observation of the patterns of kinetic rates leading to each outcome using box plots of the corresponding kinetic parameter distributions (Supplementary Figure S6), revealing certain simple relationships: *i)* the kinetic rate of the first (resource uptake) reaction step is important ${k}_{1}>{l}_{1}$: cycle A wins, or $k_1<l_1$: cycle B wins); *ii)* coexistence occurs if $k_1\sim l_1$; *iii)* at the S-type scenario, all the kinetic rates ${k}_{1}$, ${l}_{1}$ and ${k}_\text{cat}$ are high; *iv)* for the SX-type, ${k}_{1}$ and ${l}_{1}$ are low, and $k_{\text{on}_2}$ is high. Apart from these observations, an accurate explanation and interpretation of the results requires a much more complex approach, offering a possible direction to extend the present study.

## Discussion

We attempted to dive into the competitive behavior of small-molecule autocatalytic cycles. This is an important topic for the study of chemical organizations in the realm of systems chemistry, as well as for the investigations of the origins of life where chemical complexification requires dynamical coexistence of an increasing number of different chemical species.

Unsurprisingly we see parallels with the theory of the selective behavior of template replicators. There is a clear analogy between the reproductive (in our case last) step being reversible and the fact that in the case of parabolic replicators^34,35^ the dissociation of template and copy is reversible. It is this reversibility that results in parabolic (subexponential growth) that further entails dynamical coexistence in a competitive setting^36–38^. The finding that reversibility of the resource-consuming (here: first) step has a significant effect on the outcome of competition is novel, but not difficult to interpret. The reversibility of this step means that the resource is liberated so that the competitor can mop it up to benefit its own growth to the extent that the outcome of competition is reversed relative to the case when resource uptake is irreversible.

We have also considered the case when one cycle catalytically helps the growth of its otherwise inferior competitor. It is not surprising that this effect can reverse the outcome of competition (it is like helping one's enemy). Note that such an “altruistic” act also entails the price that the cycle intermediate helping the competitor is sequestered in the catalytic complex and is, therefore, unavailable for fostering the growth of its own cycle. If the helper happens to be the intermediate that is reacting with the resource the price paid is even higher. The chance for coexistence is modest indeed. The cycle being helped is in effect a parasite on the helper. The analogies with population dynamics are not accidental; the major difference between the two fields is that biological reproduction is an irreversible “reaction” (see also^39^).

This leaves us wondering whether complexification through the interactions of autocatalytic cycles is possible and to what extent. The crucial omission in this paper is the fact that the cycle being helped can also feed back positively on the helper. Such coupling can rest on different topologies^40^ with possibly qualitatively different outcomes. This is a subject of forthcoming work.

We have shown that a deep learning algorithm can be trained to predict the outcome of competition from cycle topologies and kinetic rate constants, provided the training set is large enough. This result is likely to be useful in the search for novel autocatalytic systems by complementing rule-based generative chemistry^41^.

## Methods

The general reaction scheme for an *n*-membered autocatalytic cycle consists of three types of reactions: ${\text{A}}_{1}+\text{X}{\underset{k^\prime_1}{\stackrel{{k}_{1}}{\leftrightarrow }}\text{A}_2}$ (*the resource uptake step*), ${\text{A}}_{i}\underset{k^\prime_i}{\stackrel{{k}_{i}}{\leftrightarrow }}{\text{A}}_{i+1}$ (*intermediate steps*, $2\le i<n$) and ${\text{A}}_{n}\underset{k^\prime_n}{\stackrel{{k}_{n}}{\leftrightarrow }}2{\text{A}}_{1}$ (*doubling step*), where $\text{X}$ denotes the resource, $\text{A}_i$ are the internal compounds of the autocatalytic cycle $\text{A}$, and ${k}_{i}$ and $k^\prime_{i}$ are the forward and reverse kinetic rate constants of the reactions, respectively (Fig. 1A).

We have examined the dynamics of autocatalytic cycles in two different resource supply regimes*: i) resource-unlimited systems* (Fig. 1B) and *ii)* resource-limited systems in a chemostat setting with a constant resource supply, referred to as the *chemostat system* (Fig. 1C). For the resource-unlimited system, the concentration $[\text{X}]$ of the resource is kept constant ($\text{d}\left[\text{X}\right]/\text{d}t=0$), representing an infinite external pool and an infinitely fast replenishment rate of the resource. For the chemostat system, we assume that the concentration of the resource in the inflow is $\left[\hat{\text{X}}\right]$, the inflow volume of the solution of $\text{X}$ per unit of time is $\nu$, and thus the replenishment rate of the resource is $\frac{\nu }{V}\left[\hat{\text{X}}\right]$, where $V$ is chemostat volume ($V=1, \upsilon =0.02$). Apart from the solvent, no other molecular species enters the system. Thus, the dilution rate in the chemostat is $\frac{\nu }{V}$. The outflow rate of any molecular species is proportional to the dilution rate and its present concentration: $\frac{\nu }{V}\left[{\text{A}}_{i}\right]$, cf. (Fig. 1C).

We investigate the dynamics pertaining to different scenarios by numerically integrating the corresponding set of differential equations until they reach a steady state. Deep learning algorithms trained on the dynamical system data were applied to predict qualitative outcomes.

In the first part of this study, we investigate how the dynamics of a single autocatalytic cycle depend on its resource supply regime. First, we assume an infinitely large resource pool with an infinitely fast resupply rate, i.e., the unlimited resource supply scenario is applied as a dynamical reference case, and the ensuing kinetics of the members of the cycle in qualitative and quantitative terms are characterized under different assumptions with regard to the kinetic parameters of the system. Next, we model chemostat conditions with constant throughput (as a more realistic but still analytically tractable system) to analyse the quantitative and qualitative effects on system dynamics of changing the kinetic rates of the individual reaction steps and the corresponding reverse reactions of the cycle.

In the second part, we investigate the possibility of coexistence for two competing autocatalytic cycles feeding on a single common limiting resource. The problem we are considering here may seem ecological: the possibility of coexistence for two (or more) species of reproducing entities using a common resource, which is the stereotypical situation of resource competition in ecology. The analogy, however, works only in the case of completely irreversible autocatalytic cycles, as mother and daughter cannot fuse back in biology, but the reversibility of the split reaction of a molecule into two others is possible in chemistry, with important dynamical consequences. We look for conditions of stable coexistence between two competing autocatalytic cycles, either considering or disregarding reverse reactions in one or both (dominant and/or inferior) cycles. The analysis of the effect of assuming reversible reactions separately in each of the two cycles is necessary as competitive asymmetry plays a crucial role in determining the dynamics of their interaction.

Besides competing for the same resource, two autocatalytic cycles can interact by *cross-catalysis*. In this case, a member of one cycle ($\text{A}_j$) catalyzes a reaction of the other cycle (${\text{B}}_{p}+\text{X}\underset{l^\prime_p}{\stackrel{{l}_{p}}{\leftrightarrow }}{\text{B}}_{p+1}$) following Michaelis–Menten type kinetics. Catalysis may act on the first, bimolecular reaction of the recipient cycle B ($p=1$), in which case the reaction scheme depends on the order of educt association; the two possible cases will be referred to as SX-type (substrate then resource) and XS-type (resource then substrate) catalysis. Catalysis acting on a later, monomolecular step (${\text{B}}_{p}\underset{l^\prime_p}{\stackrel{{l}_{p}}{\leftrightarrow }}{\text{B}}_{p+1}$, if $p>1$) will be called S-type). Using a deep learning algorithm trained on the data produced by our kinetic studies, we derive a method for predicting the coexistence of two autocatalytic cycles with high accuracy in any interaction scenario. See Supplementary Information for further details on the model and analysis.

### Supplementary Information

Supplementary Information.

## Data Availability

The code of the simulation and the data are available at https://zenodo.org/records/10560157.
