# Supplementary material for: Kinetics and coexistence of autocatalytic reaction cycles
Source: Sci Rep. 2024 Aug 8;14:18441. doi: 10.1038/s41598-024-69267-w (PMC11310475; doi:10.1038/s41598-024-69267-w)
Supplement: Supplementary file 1 — Supplementary Information. [file 41598_2024_69267_MOESM1_ESM.docx]

Supplementary Information

**Kinetics and Coexistence of Autocatalytic Reaction Cycles**

Balázs Könnyű^1^, Eörs Szathmáry^1,2^, Tamás Czárán^1,*^, András Szilágyi^1^

^1^HUN-REN Centre for Ecological Research, Institute of Evolution, Konkoly-Thege Miklós út 29-33. 1121 Budapest, Hungary

^2^Center for Conceptual Foundations of Science, Parmenides Foundation, Hindenburgstr. 15. 82343 Pöcking, Germany

^*^Corresponding author: Tamás Czárán

Email: czaran.tamas@ecolres.hu

**Table of Contents**

| 1. | Kinetic Equations of the Models | 2 |
| --- | --- | --- |
| 2. | Analytic Approaches to the Kinetic Equations: Single Autocatalytic Cycle without Resource Limitation | 5 |
| 3. | Numerical Analysis of the Kinetic Equations: Two (or More) Autocatalytic Cycles Competing for a Single Common Resource in the Chemostat | 9 |
| 4. | Steady State Classification Using Deep Learning | 13 |

**1 Kinetic Equations of the Models**

The general reaction scheme for an *n*-membered and an *m*-membered autocatalytic cycle using the same resource X is the following:

|  | $\begin{matrix} \begin{matrix} A_{1}+X{\underset{k_{1}^{'}}{\underset{\leftrightarrow}{k_{1}}}A}_{2} \\ A_{2}\underset{k_{2}^{'}}{\underset{\leftrightarrow}{k_{2}}}A_{3} \\ A_{3}\underset{k_{3}^{'}}{\underset{\leftrightarrow}{k_{3}}}A_{4} \\ \vdots\\ A_{n-1}\underset{k_{n-1}^{'}}{\underset{\leftrightarrow}{k_{n-1}}}A_{n} \\ A_{n}\underset{k_{n}^{'}}{\underset{\leftrightarrow}{k_{n}}}2A_{1} \end{matrix} & \mathrm{and} & \begin{matrix} B_{1}+X{\underset{l_{1}^{'}}{\underset{\leftrightarrow}{l_{1}}}B}_{2} \\ B_{2}\underset{l_{2}^{'}}{\underset{\leftrightarrow}{l_{2}}}B_{3} \\ B_{3}\underset{l_{3}^{'}}{\underset{\leftrightarrow}{l_{3}}}B_{4} \\ \vdots\\ B_{m-1}\underset{l_{m-1}^{'}}{\underset{\leftrightarrow}{l_{m-1}}}B_{m} \\ B_{m}\underset{l_{m}^{'}}{\underset{\leftrightarrow}{l_{m}}}2B_{1} \end{matrix} \end{matrix}$ | (S1a-b) |
| --- | --- | --- |

where $k_{i}$ and $k_{i}^{'}$ are the rate constants for the forward and backward reactions between $A_{i}$ and $A_{i+1}$, respectively (and between $B_{i}$ and $B_{i+1}$ in the second cycle with rate constants $l_{i}$ and $l_{i}^{'}$, Figure 1A). The kinetic equations for cycle A are the following:

|  | $\begin{matrix} \frac{d[A_{1}]}{dt}=2k_{n}\left[ A_{n} \right]-k_{1}\left[ A_{1} \right]\left[ X \right]+k_{1}^{'}\left[ A_{2} \right]-2k_{n}^{'}\left[ A_{1} \right]^{2} \\ \frac{d\left[ A_{2} \right]}{dt}=k_{1}\left[ A_{1} \right]\left[ X \right]-k_{1}^{'}\left[ A_{2} \right]-k_{2}\left[ A_{2} \right]+k_{2}^{'}\left[ A_{3} \right] \\ \frac{d\left[ A_{i} \right]}{dt}=k_{i-1}\left[ A_{i-1} \right]-k_{i-1}^{'}\left[ A_{i} \right]-k_{i}\left[ A_{i} \right]+k_{i}^{'}\left[ A_{i+1} \right] \\ \frac{d\left[ A_{n} \right]}{dt}=k_{n-1}\left[ A_{n-1} \right]-k_{n-1}^{'}\left[ A_{n} \right]-k_{n}\left[ A_{n} \right]+k_{n}^{'}\left[ A_{1} \right]^{2} \end{matrix}.$ | (S2) |
| --- | --- | --- |

The kinetic equations for cycle B are, *mutatis mutandis*, the same as Eqs. (S2).

Next, we construct the ODE systems for single-cycles and for two interacting cycles, each in two dynamical scenarios: *i)* resource-unlimited systems (Figure 1B) and *ii)* resource-limited systems in a chemostat with a constant resource supply (Figure 1C) (Schuster, P, 2019).

road

*The resource-unlimited system*

In the unlimited regime the kinetics of resource X is simple: $\frac{d[X]}{dt}=0$, i.e., the (non-zero) concentration of the resource in the system remains constant, regardless of the variable speeds of resource consumption by the autocatalytic cycle(s). Recall that this assumption implies postulating an infinite external resource pool with an infinite replenishment rate of the resource (the *resource-unlimited model;* see Figure 1B).

*The resource-limited (chemostat) system)*

The other regime in which the behavior of the autocatalytic cycle(s) was analyzed is the chemostat, with a constant-rate in- and outflow of the resource and the molecular species belonging to the cycles. The volume $V$ of the chemostat is assumed to be constant (Schuster, P, 2019). The inflow volume of a solution of components $A_{i}, B_{j}$ and resource $X$ into the chemostat in a unit of time is $\nu$ (Figure 1C). The concentrations of these chemical species in the incoming solution are $[\hat{A}_{i}]$, $[\hat{B}_{j}]$ and $[\hat{X}]$). In all the cases presented in this study, we let $[\hat{A}_{i}]= [\hat{B}_{i}] =0$, so that it is only the resource $X$ that is replenished in the chemostat. The outflow volume per unit time is also $\nu$, with component concentrations $\left[ A_{i} \right], \left[ B_{j} \right]$ and $\left[ X \right]$ in the outgoing solution. The dynamics of the component concentrations are governed by their net fluxes through the chemostat, i.e., by the differences between the concentration of substance $A_{i}$, $B_{j}$ or $X$ in the inflow ($[\hat{A}_{i}] , [\hat{B}_{j}]$ or $[\hat{X}]$) and its actual concentration ($\left[ A_{i} \right], \left[ B_{j} \right]$, or $\left[ X \right]$) within the system (and thus in the outflow), multiplied by the dilution rate $\frac{\nu}{V}$. The net fluxes are added to the corresponding reaction terms of Eq. (S2) (the original *rhs* of which are represented by triple dots):

|  | $\begin{matrix} \frac{d[A_{1}]}{dt}=\ldots+\frac{\nu}{V}\left( \left[ \hat{A}_{1} \right]-[A_{1}] \right) \\ \frac{d\left[ A_{2} \right]}{dt}=\ldots+\frac{\nu}{V}\left( \left[ \hat{A}_{2} \right]-[A_{2}] \right) \\ \frac{d\left[ A_{i} \right]}{dt}=\ldots+\frac{\nu}{V}\left( (\left[ \hat{A}_{i} \right]-[A_{i}] \right) \\ \frac{d\left[ A_{n} \right]}{dt}=\ldots+\frac{\nu}{V}\left( \left[ \hat{A}_{n} \right]-\left[ A_{n} \right] \right) , \end{matrix}$ | (S3a) |
| --- | --- | --- |

with, of course, a similar set of equations for cycle $B$. In the chemostat regime, the kinetics of the resource $X$ is quite different from that in the unregulated regime:

|  | $\frac{d\left[ X \right]}{dt}={-k}_{1}\left[ A_{1} \right]\left[ X \right]+k_{1}^{'}\left[ A_{2} \right]{-l}_{1}\left[ B_{1} \right]\left[ X \right]+l_{1}^{'}\left[ B_{2} \right]+\frac{\nu}{V}\left( \left[ \hat{X} \right]-\left[ X \right] \right).$ | (S3b) |
| --- | --- | --- |

Under these assumptions, the total growth rate of the pooled concentrations of autocatalytic cycles $A$ and $B$ is limited (regulated) by the finite replenishment rate $\frac{\nu}{V}\left[ \hat{X} \right]$ of their common resource $X$ (Figure 1C).

*Cross-catalysis*

The kinetic interaction of the two autocatalytic cycles through their common resource $X$ (which occurs if it is of limited supply) is indirect. We allow for the possibility of the cycles directly affecting each other’s kinetics by the catalysis of one or another reaction of the other cycle. To include cross-catalysis, we assume Michaelis–Menten type kinetics with rate constants $k_{\mathrm{on}}$, $k_{\mathrm{off}}$ and $k_{\mathrm{cat}}$: one member of cycle $A$ (say, $A_{j}$) catalyzes a reaction step in cycle $B$ (say, the *p*^th^ reaction producing $B_{p+1}$ from $B_{p}$, green arrows in Figure 1A). The reaction scheme of cross-catalysis depends on which of the reactions of the catalysed cycle is the target. If $p>1$, the catalysed reaction is monomolecular (hereafter S-type), thus the reaction scheme of the cross-catalytic step is the following:

|  | $A_{j}+B_{p}\underset{k_{\mathrm{off}}}{\underset{\leftrightarrow}{k_{\mathrm{on}}}}A_{j}B_{p}\underset{\to}{k_{\mathrm{cat}}}A_{j}+B_{p+1}$ (S-type). | (S4a) |
| --- | --- | --- |

If $p=1$, the catalysed reaction is bimolecular; for example, the $B_{1}+X{\underset{l_{1}^{'}}{\underset{\leftrightarrow}{l_{1}}}B}_{2}$ reaction is catalysed by $A_{j}$. The actual reaction scheme depends on the association order of the educts (the resource molecule $X$ and the substrate $B_{1}$) to the catalyst, hereafter SX-type (substrate then X) and XS-type (X then substrate):

|  | $A_{j}+B_{1}\underset{{k_{\mathrm{off}}}_{1}}{\underset{\leftrightarrow}{k_{\mathrm{on}_{1}}}}A_{j}B_{1}, A_{j}B_{1}+X\underset{{k_{\mathrm{off}}}_{2}}{\underset{\leftrightarrow}{k_{\mathrm{on}_{2}}}}A_{j}B_{1}X\underset{\to}{k_{\mathrm{cat}}}A_{j}+B_{2}$ (SX-type) | (S4b) |
| --- | --- | --- |
|  | $A_{j}+X\underset{{k_{\mathrm{off}}}_{1}}{\underset{\leftrightarrow}{k_{\mathrm{on}_{1}}}}A_{j}X, A_{j}X+B_{1}\underset{{k_{\mathrm{off}}}_{2}}{\underset{\leftrightarrow}{k_{\mathrm{on}_{2}}}}A_{j}XB_{1}\underset{\to}{k_{\mathrm{cat}}}A_{j}+B_{2}$ (XS-type). | (S4c) |

We will see in the *Results* section that the actual position and stoichiometry of the catalysed reaction can make a qualitative difference in the kinetics.

In the case of a S-type cross catalysis (see Eq. (S4a)), the additive terms of the corresponding set of differential equations are the following (triple dots are placeholders for the corresponding *rhs* of Eqs. (S3a)):

|  | $\begin{matrix} \frac{d\left[ A_{j} \right]}{dt}=\ldots+\left( k_{\mathrm{off}}-k_{\mathrm{cat}} \right)\left[ A_{j}B_{p} \right]-k_{\mathrm{on}}\left[ A_{j} \right]\left[ B_{p} \right] \\ \frac{d\left[ B_{p} \right]}{dt}=\ldots+k_{\mathrm{off}}\left[ A_{j}B_{p} \right]-k_{\mathrm{on}}\left[ A_{j} \right]\left[ B_{p} \right] \\ \frac{d\left[ B_{p+1} \right]}{dt}=\ldots+k_{\mathrm{cat}}\left[ A_{j} \right]\left[ B_{p} \right] \\ \frac{d\left[ {A_{j}B}_{p} \right]}{dt}=k_{\mathrm{on}}\left[ A_{j} \right]\left[ B_{p} \right]-\left( k_{\mathrm{off}}+k_{\mathrm{cat}} \right)\left[ A_{j}B_{p} \right]+\frac{\nu}{V}\left( \left[ \hat{A_{j}B_{p}} \right]-\left[ A_{j}B_{p} \right] \right), \end{matrix}$ | (S5a) |
| --- | --- | --- |

where the last equation describes the dynamics of the ${A_{j}B}_{p}$ complex (and its net flux for chemostat systems).

In the case of SX- and XS-type cross catalysis, the additive terms of the corresponding set of differential equations (cf. Eqs. (S3b) (S4b) and (S4c), respectively) are the following.

For SX-type catalysis:

|  | $\frac{d\left[ A_{j} \right]}{dt}=\ldots-k_{\mathrm{on}_{1}}\left[ A_{j} \right]\left[ B_{1} \right]+k_{\mathrm{off}_{1}}\left[ A_{j}B_{1} \right]+k_{\mathrm{cat}}\left[ A_{j}B_{1}X \right]$ $\frac{d\left[ B_{1} \right]}{dt}=\ldots-k_{\mathrm{on}_{1}}\left[ A_{j} \right]\left[ B_{1} \right]+k_{\mathrm{off}_{1}}\left[ A_{j}B_{1} \right]$ $\frac{d\left[ B_{2} \right]}{dt}=\ldots+k_{\mathrm{cat}}\left[ A_{j}B_{1}X \right]$ $\frac{d\left[ {A_{j}B}_{1}X \right]}{dt}=k_{on_{2}}\left[ A_{j}B_{1} \right]\left[ X \right]-\left( k_{\mathrm{off}_{2}}+k_{\mathrm{cat}} \right)\left[ A_{j}B_{1}X \right]+\frac{\nu}{V}\left( \left[ \hat{A_{j}B_{1}X} \right]-\left[ A_{j}B_{1}X \right] \right)$ $\frac{d\left[ A_{j}B_{1} \right]}{dt}=k_{\mathrm{on}_{1}}\left[ A_{j} \right]\left[ B_{1} \right]-k_{\mathrm{off}_{1}}\left[ A_{j}B_{1} \right]-k_{on_{2}}\left[ A_{j}B_{1} \right]\left[ X \right]+k_{\mathrm{off}_{2}}\left[ A_{j}B_{1}X \right]+$ $+\frac{\nu}{V}\left( \left[ \hat{A_{j}B_{1}} \right]-\left[ A_{j}B_{1} \right] \right)$ $\frac{d\left[ X \right]}{dt}=\ldots-k_{on_{2}}\left[ A_{j}B_{1} \right]\left[ X \right] +k_{\mathrm{off}_{2}}\left[ A_{j}B_{1}X \right],$ | (S5b) |
| --- | --- | --- |

and for XS-type catalysis:

|  | $\frac{d\left[ A_{j} \right]}{dt}=\ldots-k_{\mathrm{on}_{1}}\left[ A_{j} \right]\left[ X \right]+k_{\mathrm{off}_{1}}\left[ A_{j}X \right]+k_{\mathrm{cat}}\left[ A_{j}B_{1}X \right]$ $\frac{d\left[ B_{1} \right]}{dt}=\ldots-k_{\mathrm{on}_{2}}\left[ A_{j}X \right]\left[ B_{1} \right]+k_{\mathrm{off}_{2}}\left[ A_{j}B_{1}X \right]$ $\frac{d\left[ B_{2} \right]}{dt}=\ldots+k_{\mathrm{cat}}\left[ A_{j}B_{1}X \right]$ $\frac{d\left[ A_{j}X \right]}{dt}=k_{\mathrm{on}_{1}}\left[ A_{j} \right]\left[ X \right]-k_{\mathrm{off}_{1}}\left[ A_{j}X \right]-k_{on_{2}}\left[ A_{j}X \right]\left[ B_{1} \right]+k_{\mathrm{off}_{2}}\left[ A_{j}B_{1}X \right]+$ $+\frac{\nu}{V}\left( \left[ \hat{A_{j}X} \right]-\left[ A_{j}X \right] \right)$ $\frac{d\left[ {A_{j}B}_{1}X \right]}{dt}=k_{on_{2}}\left[ A_{j}X \right]\left[ B_{1} \right]-\left( k_{\mathrm{off}_{2}}+k_{\mathrm{cat}} \right)\left[ A_{j}B_{1}X \right]+\frac{\nu}{V}\left( \left[ \hat{A_{j}B_{1}X} \right]-\left[ A_{j}B_{1}X \right] \right)$ $\frac{d\left[ X \right]}{dt}=\ldots-k_{on_{1}}\left[ A_{j} \right]\left[ X \right] +k_{\mathrm{off}_{1}}\left[ A_{j}X \right],$ | (S5c) |
| --- | --- | --- |

with both sets of equations extended to include the dynamics of the complexes (and their net fluxes for chemostat systems).

**2 Analytic Approaches to the Kinetic Equations: Single Autocatalytic Cycle without Resource Limitation**

Due to the complexity of the chemical kinetics, there is no analytic solution for the sets of differential equations Eqs. (S2), (S3) or (S5) in the general case. In the following we derive an analytic solution for the simplest case (single cycle, unlimited resource input, irreversible reactions, uniform kinetic constants) and show partial results for more complex systems without resource limitation.

*Cases 1: Fully irreversible cycle*

In this case, $k_{i}^{'}=0, i=1\ldots n$. To solve the corresponding set of differential equations, we use the following solution approach to the asymptotic behaviour:

|  | $\left[ A_{i} \right]=a_{i}e^{\lambda t},$ | (S6) |
| --- | --- | --- |

i.e., all members of the autocatalytic cycle grow exponentially with the same exponent. By plugging Eq. (S6) into Eq. (S2), cancelling the exponential terms and dividing the *i*th equation by $a_{i}$, after simple rearrangements we get the following set of algebraic equations:

|  | $\begin{matrix} \lambda+k_{1}\left[ X \right]=2k_{n}\frac{a_{n}}{a_{1}} \\ \lambda+k_{2}=k_{1}\frac{a_{1}}{a_{2}}\left[ X \right] \\ \lambda+k_{i}=k_{i-1}\frac{a_{i-1}}{a_{i}} \end{matrix},$ | (S7) |
| --- | --- | --- |

where $i\geq3$. Note that multiplication of two consecutive equations leads to the cancellation of $a$ terms on the right-hand sides, e.g., $\left( \lambda+k_{i} \right)\left( \lambda+k_{i+1} \right)=k_{i-1}k_{i}\frac{a_{i-1}}{a_{i+1}}$ ($a_{i}$ has been cancelled). Upon multiplying the equations of (S7), we arrive at the following form:

|  | $\left( \lambda+k_{1}[X] \right)\prod_{i=2}^{n} \left( \lambda+k_{i} \right)=2[X]\prod_{i=1}^{n} k_{i}.$ | (S8) |
| --- | --- | --- |

We can set $\left[ X \right]=1$ by using appropriate units for concentrations; then Eq. (S8) takes a simpler form:

|  | $\prod_{i=1}^{n} \left( \lambda+k_{i} \right)=2\prod_{i=1}^{n} k_{i}.$ | (S9) |
| --- | --- | --- |

This equation is an *n*th-order algebraic equation which, in the general case, can be solved numerically. The growth rate of the system is the (largest) positive root of Eq. (S9). After some rearrangement we obtain the following formula:

|  | $\lambda^{n}+\lambda^{n-1}\sum_{\{i\}} k_{i}+\lambda^{n-2}\sum_{\{i,j\}} k_{i}k_{j}+\lambda^{n-3}\sum_{\{i,j,l\}} k_{i}k_{j}k_{l}+\ldots+\lambda\sum_{\left\{ i,j,l,\ldots\right\}} k_{i}k_{j}k_{l}\ldots-\prod_{i=1}^{n} k_{i}=0$ | (S10) |
| --- | --- | --- |

where $\{i,j\}$ denotes all distinct pairs, $\{i,j,l\}$ denotes all distinct triplets, …, $\left\{ i,j,l,\ldots\right\}$ denotes all distinct ($n-1$)-tuplets from the $\{1,2,\ldots,n\}$ set of integers. As the number of sign changes in the sequence of the polynomial’s coefficients is one (all *k*’s are positive), according to Descartes’ rule of signs, Eq. (S9) has one positive root, which is the growth rate of the system.

There is no general analytical solution to this equation, except for uniform rate constants $\left( k_{i}=k, i=1,\ldots,n \right)$, in which case the solution is straightforward:

|  | $\lambda=\left( \sqrt[n]{2}-1 \right)k.$ | (S11) |
| --- | --- | --- |

*Case 2: Reversible reaction(s) on any internal step, irreversible doubling step*

In this case, one or more internal reaction steps are reversible, ie., $\sum_{i=1}^{n-1} k_{i}^{'}>0$, but the last reaction step is irreversible: $k_{n}^{'}=0.$ Applying the previous solution approach we get the following set of equations:

|  | $\lambda a_{1}=-k_{1}a_{1}\left[ X \right]+k_{1}^{'}a_{2}+2k_{n}a_{n}$ $\lambda a_{2}=k_{1}a_{1}\left[ X \right]-k_{1}^{'}a_{2}-k_{2}a_{2}+k_{2}^{'}a_{3}$ $\lambda a_{i}=k_{i-1}a_{i-1}-k_{i-1}^{'}a_{i}-k_{i}a_{i}+k_{i}^{'}a_{i+1}$ | (S12) |
| --- | --- | --- |

where $i\geq3$. This can be solved numerically; the resulting (largest) positive root is the growth rate of the autocatalytic cycle. (Because of the complexity of the equations, Descartes' rule of signs cannot be applied here.) System growth remains exponential, but its overall growth rate is smaller than the growth rate of the corresponding irreversible cycle (see Table 1 in the main text).

*Case 3: Reversible reaction at the doubling step; cycle not fully reversible*

In this case, $k_{n}^{'}\neq0$, and at least one intermediate step is irreversible: $\prod_{i=1}^{n-1} k_{i}^{'}=0$, so that the cycle cannot turn in the reverse direction. The former condition implies that, after plugging the $\left[ A_{n} \right]=a_{n}e^{\lambda t}$ solution approach into the last equation of Eq. (S2), it is not possible to cancel the exponential terms, as there is an $\left[ A_{1} \right]^{2}$ term in the equation:

|  | $\lambda a_{n}=k_{n-1}a_{n-1}-k_{n-1}^{'}a_{n}-k_{n}a_{n}+k_{n}^{'}a_{1}^{2}e^{\lambda t};$ | (S13) |
| --- | --- | --- |

consequently, no constant $\lambda$ describes system growth, which, therefore, is not exponential.

Numerical analysis of the Eq. (S2) system of ODEs suggests that in this case the asymptotic growth of the concentration $\left[ A_{n} \right]$ of the last (doubling) member of the autocatalytic cycle is quadratic in time, while all other members grow linearly:

|  | $\left[ A_{j} \right]=a_{j}t$ $\left[ A_{n} \right]=a_{n}t^{2}$ | (S14) |
| --- | --- | --- |

where $j=1,2,\ldots,n-1$, see Figure S1. Substituting these empirical asymptotic expressions into the differential equations of Eq. (S2) yields

|  | $\begin{matrix} a_{1}=2k_{n}a_{n}t^{2}-k_{1}a_{1}t\left[ X \right]+k_{2}^{'}a_{2}t-2k_{n}^{'}a_{1}^{2}t^{2} \\ a_{2}=k_{1}a_{1}t\left[ X \right]-k_{2}a_{2}t-k_{2}^{'}a_{2}t+k_{3}^{'}a_{3}t \\ a_{i}=k_{i-1}a_{i-1}t-k_{i}a_{i}t-k_{i}^{'}a_{i}t+k_{i+1}^{'}a_{i+1}t, (i=3,\ldots,n-1) \\ 2a_{n}t=k_{n-1}a_{n-1}t-k_{n}a_{n}t^{2}+k_{n}^{'}a_{1}^{2}t^{2}-k_{n-1}^{'}a_{n-1}t. \end{matrix}$ | (S15) |
| --- | --- | --- |

To obtain time-independent identities for *a_i_* ($i=1,\ldots,n$) in the asymptotic $t\to\infty$ limit, the sum of the prefactors of the leading terms in $t$ must be zero in each of these equations. Since the first and the last equation yield the same condition, we have $n-1$ equations in $n$ unknowns ($a_{1}, a_{2},\ldots, a_{n}$), i.e., the system is underdetermined: the remaining $n-1$ independent identities define only pairwise ratios for the parameters $a_{i}$ ($i=3,\ldots,n-1$):

$$\begin{matrix} \frac{a_{1}^{2}}{a_{n}}=\frac{k_{n}}{k_{n}^{'}} \\ \frac{a_{1}}{a_{2}}=\frac{1}{\left[ X \right]}\frac{k_{2}}{k_{1}} \\ \frac{a_{i-1}}{a_{i}}=\frac{k_{i}}{k_{i-1}} \end{matrix}$$

**
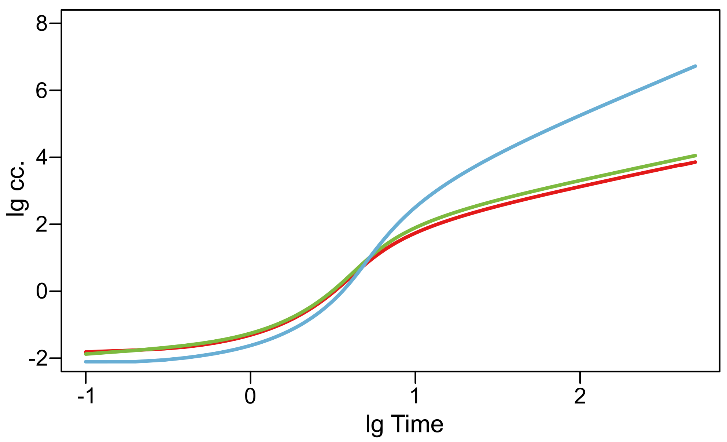
**

**Figure S1.** The time course of the concentration of a 3-membered cycle. Reversible reaction at the doubling step; cycle not fully reversible (*Case 3*), rate constants: $k_{1}=5.87, k_{2}=3.84, k_{3}=7.56$ and $k_{3}^{'}=0.76$. Concentrations of the members $A_{1},A_{2}$ $A_{3}$ are shown as red, green, and blue curves, respectively. Note that both axes are logarithmic, and the initial concentrations were $\left[ A_{1} \right]\left( 0 \right)=\left[ A_{2} \right]\left( 0 \right)=\left[ A_{3} \right]\left( 0 \right)=0.01$.

*Case 4:* *Reversible reactions at all steps (fully reversible cycle)*

|  | $\begin{matrix} k_{n}a_{n}=k_{n}^{'}a_{1}^{2} \\ k_{1}a_{1}\left[ X \right]+k_{3}^{'}a_{3}=\left( k_{2}+k_{2}^{'} \right)a_{2} \\ \left( k_{i}+k_{i}^{'} \right)a_{i}=k_{i-1}a_{i-1}+k_{i+1}^{'}a_{i+1} (i=3,\ldots,n-1) \end{matrix}$ | (S16) |
| --- | --- | --- |

In this case, $k_{i}^{'}>0, i=1,\ldots,n$. With all the reactions of the autocatalytic system reversible, the reaction cycle can turn in the forward as well as the backward direction, allowing for the self-regulation of the system that, consequently, admits a stable fixed point. This can be calculated from Eq. (S2) by setting its *rhs* equal to 0, which yields equilibrium concentrations for the members of the cycle as

|  | $\left[ \bar{A}_{1} \right]=\left[ X \right]\frac{\prod_{j=1}^{N} k_{j}}{\prod_{j=1}^{N} k_{j}^{'}}$ | (S17) |
| --- | --- | --- |
|  | $\left[ \bar{A}_{i} \right]=\left[ X \right]^{2}\frac{\prod_{j=1}^{i-1} k_{j}^{2}\prod_{j=i}^{N} k_{j}}{\prod_{j=1}^{i-1} k_{j}^{'2}\prod_{j=i}^{N} k_{j}^{'}}$ | (S18) |

Note that with any of the reactions irreversible ($\prod_{i=1}^{n} k_{i}^{'}=0)$, this formula diverges, corresponding either to exponential (if $k_{n}^{'}=0)$ or subexponential (if $k_{n}^{'}\neq0 \mathrm{and} \prod_{i=1}^{n-1} k_{i}^{'}=0$) growth.

**
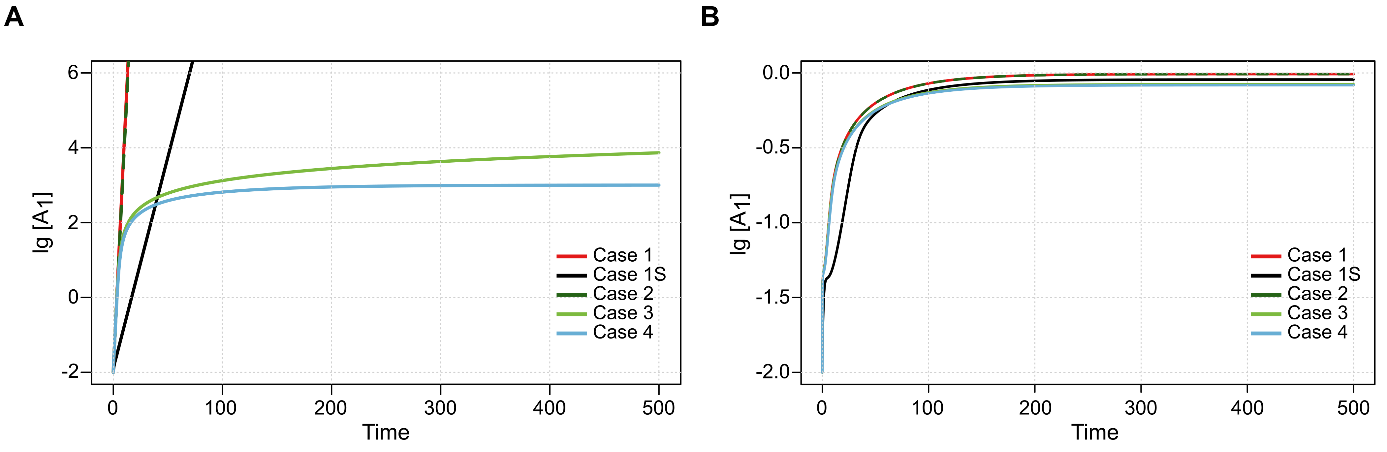
**

**Figure S2.** Time courses of the concentration of the first element ($[A_{1}]$) of the autocatalytic cycle in the discussed four scenarios. A. Dynamics of resource-unlimited systems. Red curve (Case 1): no reverse reaction ($k_{1}=5.87, k_{2}=3.84, k_{3}=7.56$), $\lambda_{1}=0.606$; black curve (Case 1S): no reverse reactions, the rate constants are identical ($k_{1}=1.00, k_{2}=1.00, k_{3}=1.00$), $\lambda_{1s}=0.113$; dashed green curve (Case 2): the first reaction step is reversible ($k_{1}^{'}=0.59$, all other rates are as before), $\lambda_{2}=0.586$; light green curve (Case 3): the last (doubling) reaction step is reversible ($k_{3}^{'}=0.76$, all other rates are as in Case 1); blue curve (Case 4): all reactions are reversible ($k_{1}^{'}=0.59, k_{2}^{'}=0.38, k_{3}^{'}=0.76,$ all other rates are as in Case 1). Note the logarithmic *y*-axis. B. Chemostat systems. The rate constants and colors are the same as in panel A. In the resource-unlimited simulations the concentration of the resource was fixed at $\left[ X \right]=1.0$; the initial concentrations in all simulations were $\left[ A_{1} \right]\left( 0 \right)=\left[ A_{2} \right]\left( 0 \right)=\left[ A_{3} \right]\left( 0 \right)=0.01.$

**3 Numerical Analysis of the Kinetic Equations: Two (or More) Autocatalytic Cycles Competing for a Single Common Resource in the Chemostat**

Since there is no general analytic solution, to the chemical kinetics, we have used numerical methods to analyse the behaviour of different systems, by integrating the corresponding set of differential equations (Eqs. (S2), (S3) or (S5)). In the case of exponential growth (resource-unlimited models), the growth rate of a cycle can be approximated by numerical integration, as the slope of the line fitted on the logarithmic concentrations of its first element ($A_{1}$) versus time. In case of a steady-state equilibrium (in the chemostat regime) the integration is terminated if the absolute value of all of the relative differences of concentrations $A_{i}$ (and $B_{i}$) at $t$ and $t+\Delta t$ is smaller than ${10}^{-6}$ ($\Delta t=1000$), that is, if $\frac{|{[A]}_{i}\left( t+\Delta t \right)-{[A]}_{i}\left( t \right)|}{{[A]}_{i}\left( t+\Delta t \right)}<{10}^{-6}$ for all $i$; likewise for cycle $B$. We consider an autocatalytic cycle extinct if the sum of the concentrations of its elements drops below ${10}^{-6}$, i.e., $\sum_{i=1}^{n} [A_{i}]<{10}^{-6}$ or $\sum_{i=1}^{m} [B_{i}]<{10}^{-6}$.

All numerical integrations were performed in C using the Sundials CVODE 5.7.0 package (Gardner et al., 2022).

A thorough analysis of the dynamics of two autocatalytic cycles requires a solution of the corresponding system of ordinary differential equations, Eqs. (S3a) and (S3b). Even though the full analytic treatment of this ODE system is even less doable than that of single-cycle systems, a numerical analysis of the dynamics is still possible, but it has to cover the whole parameter space of the model, which is the direct product of the feasible ranges of all kinetic constants ($k_{i},k_{i}^{'},l_{j},l_{j}^{'}, i=1\ldots n, j=1\ldots m$) of Eq. (S3) and also $k_{\mathrm{on}}, k_{\mathrm{off}}, k_{\mathrm{cat}}$in case of Eqs. (S5) . The computational capacity that a consistent scanning of this space would require is prohibitively high, therefore we used the Monte-Carlo method to sample the parameter space of the system. Specifically, we have drawn a large number (${10}^{5}$) of independent sets of forward kinetic rates (*k-* and *l-sets,* hereafter *{k,l}-sets*) at random from the (0.1, 10.0) interval for the two cycles ($k_{i},l_{j}\in U\left( 0.1, 10.0 \right), i=1\ldots n, j=1\ldots m$) and recorded the outcomes (steady states) of the corresponding ODEs.


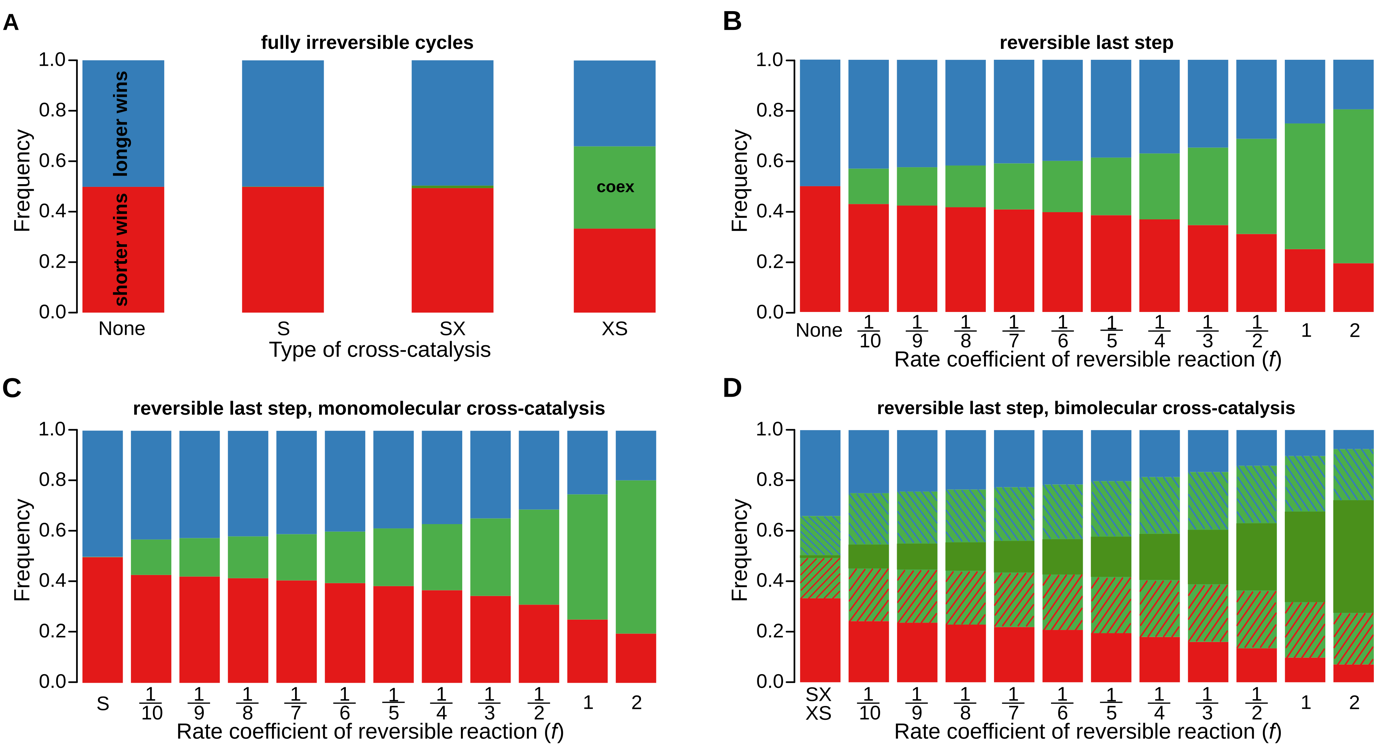


**Figure S3.** Coexistence and exclusion between two cycles of the same lengths in a chemostat. Cycles $A$and B have 3 members ($n=m=3$). Red: cycle A wins; blue: cycle B wins; green: the two cycles coexist. A: Frequencies of different outcomes of the simulations in case of fully irreversible cycles. Bar ‘None’: no cross-catalysis; bar ‘S’: monomolecular cross-catalysis; bar ‘SX’: bimolecular ‘substrate than resource’ cross-catalysis; bar ‘XS’: bimolecular ‘resource than substrate’ cross-catalysis based. B: The effect of the rate coefficient ($f$) of the reverse reaction on the last step of the dominant cycle. C: The joint effect of a reverse reaction on the last step of the dominant cycle and monomolecular cross-catalysis. D: The effect of a reverse reaction on the last step of the dominant cycle and both types of bimolecular cross-catalytic interaction (SX and XS). Solid and crosshatched red and blue boxes represent the proportion of cases (i.e., {*k,l*}-sets) in which the shorter cycle and the longer cycle wins in the SX scenario, respectively. Dark green boxes show the proportions of the coexistent cases in the SX scenario. Full blue, red and green (light green behind the crosshatched and dark green boxes together) represent the proportion of wins for the shorter cycle, for the longer cycle, and the coexistence of the two cycles, in the XS scenario, respectively. In all scenarios we ran ${10}^{5}$ independent Monte Carlo simulations with random {$k, l$}-sets. Initial concentrations: ${[A}_{i}]{=[B}_{j}]=0.01$ for all $i$ and $j$, the resource supply is $\left[ \hat{X} \right]=1.0$.$\nu=0.02, V=1.0$.

*The effect of a single reversible reaction in the dominant cycle:*

Figures 3, S4 and S5 show the results of the numerical solutions of the ODE systems with a single reverse reaction at different positions of the dominant cycle. In Figure 3 each row of plots corresponds to the position of the reverse reaction within the dominant cycle (top to bottom: 1^st^, 2^nd^ and 3^rd^ in left panels, with cycle A as the dominant, and 1^st^, 2^nd^ and 7^th^ in right panels, with cycle B as the dominant). The first bar in each panel represents the outcome without reverse reaction (*f* $=0$, which is the reference case discussed in Section 4.1 always resulting in the exclusion of the inferior cycle). Each following bar from left to right corresponds to increasing reverse rate coefficients ($f$). Note that the left and right panels of the Figures 3 and S4 in the same row show the effect of the reverse reaction in the same functional position (first, intermediate and last step). Figure S5 shows the effect of increasing *f* for all internal positions (*j* = 2, …, *m*-1) with (longer) cycle $B$ as the dominant, suggesting that it does not really make a difference even in the quantitative sense which internal reaction of the dominant cycle is reversible (for clarity, the positions from the 3^rd^ to the 6^th^ are not shown in Figure 3).


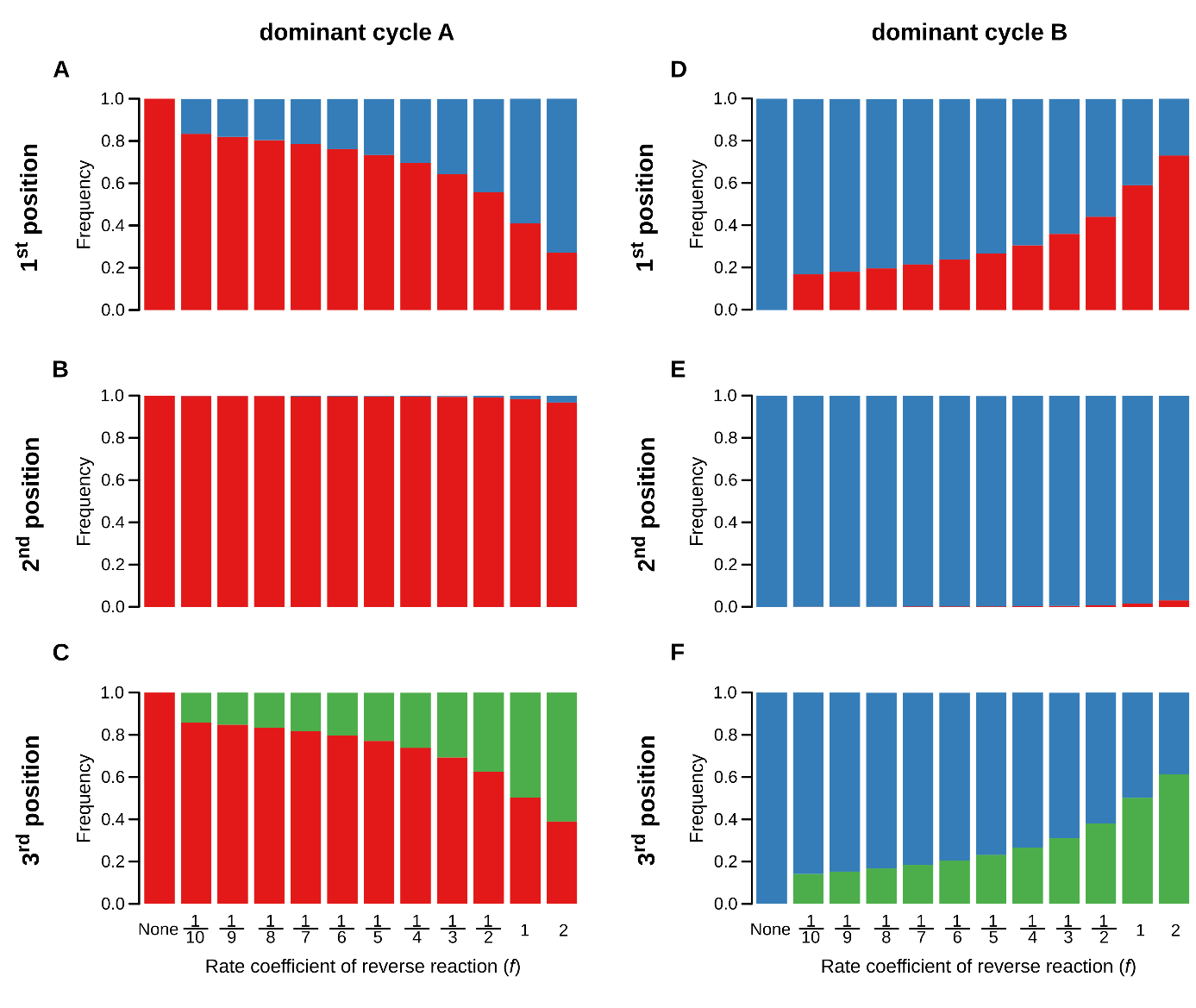


**Figure S4.** Effect of the position and the reverse rate coefficient ($f$) of a single reversible reaction in the dominant cycle. Cycle A and B have the same length ($n=m=3$. Left panels (A, B and C) show the effect of the reverse reaction at the 1^st^, 2^nd^ and 3^rd^ reaction step, respectively, when the dominant cycle is $A$. Right panels (D, E and F) show the effect of the reverse reaction at 1^st^, 2^nd^ and 3^nd^ -reaction step, respectively, when the dominant cycle is $B$. In each panel, the reverse rate coefficient increases from left to right. Red: cycle $A$wins, blue: cycle $B$wins, and green: cycles $A$and $B$ coexist. In all scenarios we ran ${10}^{5}$ independent simulations with random parameter sets. Initial concentrations: ${[A}_{i}]{=[B}_{j}]=0.01$ for all $i$ and $j$, the resource supply is $\left[ \hat{X} \right]=1.0$.$\nu=0.02, V=1.0$.


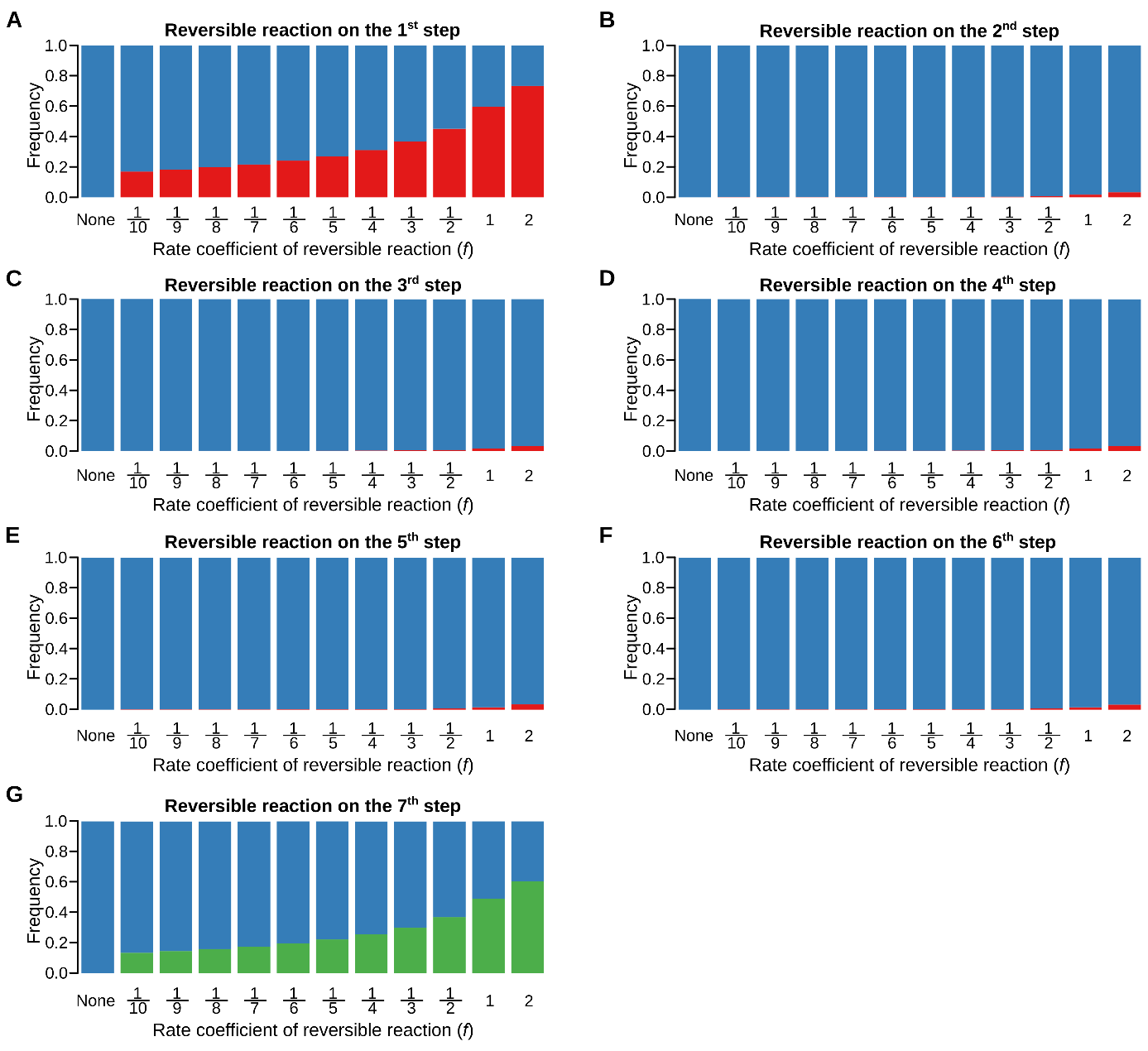


**Figure S5.** Effect of the position and the rate constant of reverse reactions (*f*) in the dominant 7-step cycle B. This is the complete version of the right column of plots in Figure 3, with all the possible positions of the reverse reaction represented, not just the first two and the last. In each panel, the rate coefficient of the reverse reactions ($f$) increased from left to right Red: cycle $A$wins; blue: cycle $B$wins; and green: cycles $A$and $B$ coexist. In all scenarios ${10}^{5}$ independent simulations with random parameter sets were accomplished. Initial concentrations: ${[A}_{i}]{=[B}_{j}]=0.01$ for all $i$ and $j$, the resource supply is $\left[ \hat{X} \right]=1.0$.$\nu=0.02, V=1.0$.

*Cross-catalysis between competing autocatalytic cycles*

Besides the indirect (competitive) interaction of the two autocatalytic cycles $A$ and $B$, which is mediated by their single common resource $X$, we also assume a direct catalytic interaction between them (see Eqs. (S4a-c)). From the viewpoint of the two cycles’ coexistence, such catalytic help may be effective only if the dominant cycle helps the slower one. In order to implement this, a random member $A_{j}$ of the dominant cycle is chosen as the catalyst that helps the formation of a random member $B_{p+1}$ of the other (competitively inferior) cycle (see Eq. (S4a) for $p>1$ and Eqs. (S4b-c) for $p=1$). The rate constants of the Michaelis—Menten reaction $k_{{on}_{1}}$ and/or $k_{{on}_{2}}$ , as well as $k_{cat}$, are drawn at random from the uniform distribution on the [0.1,10.0] interval, and $k_{off_{1}}=hk_{on_{1}}$as well as $k_{\mathrm{off}_{2}}={hk}_{{on}_{2}}$, where $h\in U[0.1, 0.5]$, and $k_{\mathrm{cat}}^{'}=0.0$. Note that both types of educt association order: SX-type (substrate then resource X) and XS-type (resource X then substrate) were investigated in the case of *p* = 1.

The ‘S’ columns in Figures 2A and S3A show the effect of monomolecular cross-catalysis (S-type) on the coexistence of cycles $A$ and $B$. The catalytic help provided by the dominant cycle imposes a handicap on the dominant cycle itself, as its turning speed decreases as its catalytically active member is engaged in catalysing a reaction of the inferior cycle, so that the competitively inferior cycle may become dominant and exclude the original dominant. If the catalyst is the first (resource capturing) member of the dominant cycle, this handicap becomes double, because a fraction of the resource-capturing species is occupied as a catalyst helping the inferior cycle, thus reducing the flow of the resource X into the original dominant cycle. Cycle length and competitive edge (the original difference between the effective growth rates of the competing cycles) impact these effects: shorter and/or less inferior catalyzed cycles are more likely to become dominant.

In the SX-type (substrate than resource X) and XS-type (resource X than substrate) scenarios without reverse reactions allowed (i.e., both in Figures 2A and S3A, bars ‘SX’ and ‘XS’) with a catalytically active member of the dominant cycle helping the resource-capturing bimolecular reaction of the inferior cycle, the effects of SX-type and XS-type reactions on the coexistence of the two cycles are qualitatively different: XS-type may lead to coexistence, but SX-type cannot. The explanation for this peculiar difference in the outcomes must lie in a division of the resource X between the two cycles in a regulated manner in XS-type and the lack thereof in SX-type, but the precise mechanism of the regulation is not yet clear, depending on the reaction rates.

The possible coexistence-promoting effect of the combination of a reversible last step in the dominant cycle and the cross catalytic aid given by the dominant cycle are summarized in Figures 2 and S3. In the cases of cross-catalysis combined with reverse reactions we see that monomolecular catalysis (S-type) has no substantial effect on coexistence (bar ‘S’ in Figures 2C for different cycle lengths and S3C for equal lengths), while the “substrate then X” mechanism of bimolecular catalysis (SX-type) appears to be detrimental, whereas the “X then substrate” (XS-type) mechanism is beneficial (Figures 2D and S3D, bars ‘SX’ and ‘XS’) for the propensity for the coexistence of reversible cycles.

**4 Steady State Classification Using Deep Learning**

We have constructed neural networks in *PyTorch* to classify the dynamical outcomes of competition between two resource-regulated autocatalytic cycles for scenarios of the chemostat model (c.f. section 4): fully irreversible cycles (section 4.1), cycles with a reverse reaction at the duplicating steps (section 4.2), and cycles with the three cross-catalytic relationships S-, SX-, and XS-type (section 4.3). Our primary goal was to predict coexistence at the fixed points of the corresponding ODE systems with independently generated random $\{k,l\}$-sets, without the need to perform computationally resource-intensive numerical integration. To obtain a sufficiently large pool of data for the neural network classifications, we numerically integrated 5.5 million competing cycles with different $\{k,l\}$-sets. The training sets were sampled from this pool. The sizes of the two competing cycles were equal ($n=m=3$) for all scenarios studied. Different neural networks were developed for each scenario (see the *.ipynb* files in the SI material) which had the following common basic structure: *i)* two linear layers with the ReLU activation function, *ii)* the number of hidden neurons was 32 , *iii)* the input parameters were the kinetic rates. This list was extended by adding the identities of the catalyst and the catalyzed reaction in the cross-catalytic scenarios, *iv)* the output was a one-dimensional binary vector characterizing the outcomes ($\{1,0,0\}$: cycle A wins, $\{0,1,0\}$: cycle B wins, $\{0,0,1\}$: coexistence of the two cycles), and *v)* the training set was divided into a training and a validation subset in a 0.8-0.2 ratio. We have trained the whole network for 10 epochs by optimizing the Binary Cross Entropy (BCE) loss function using the SGD (Stochastic Gradient Descent) optimizer and the OneCycle learning rate (with a maximal learning rate value of 0.01) scheduler. Neural networks classify most effectively with equal sample sizes for each possible outcome in the training set, so we adjusted the sample sizes accordingly (Table S1). Note that for the cross-catalytic S-type and SX-type scenarios coexistence is a relatively rare occurrence, so maintaining equal sample sizes for all possible outcomes resulted in smaller training sets for these scenarios than for others.

**Table S1** The number of outcomes in training sets.

|  | **cycle A wins** | **cycle B wins** | **cycle A and B coexist** |
| --- | --- | --- | --- |
| **None** | 150 000 | 150 000 | 0 |
| **Reverse (**$\boldsymbol{f}$**)** | 100 000 | 100 000 | 100 000 |
| **Cross-catalysis S-type** | 3 487 | 3 487 | 3 487 |
| **Cross-catalysis SX-type** | 18 667 | 18 667 | 18 667 |
| **Cross-catalysis XS-type** | 100 000 | 100 000 | 100 000 |


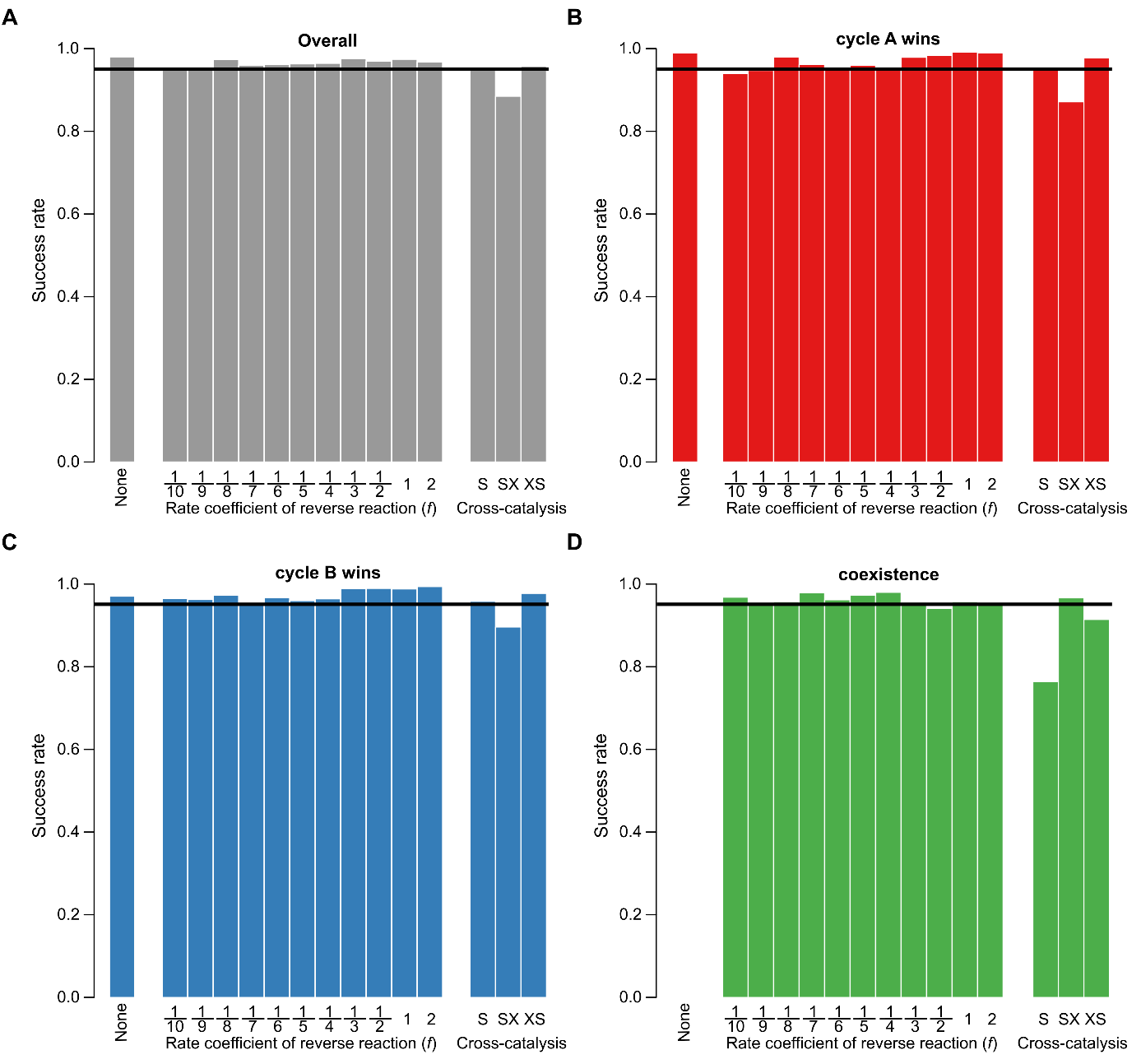


**Figure S6.** Success rates of neural networks. Panel A: the overall success rate, Panels B, C, and D show the success rates of the different outcomes: cycle A wins (red), cycle B wins (blue) and cycle A and B coexist, respectively. The black lines show the 95% success rate. The sizes of the cycles were the same: $n=m=3$.


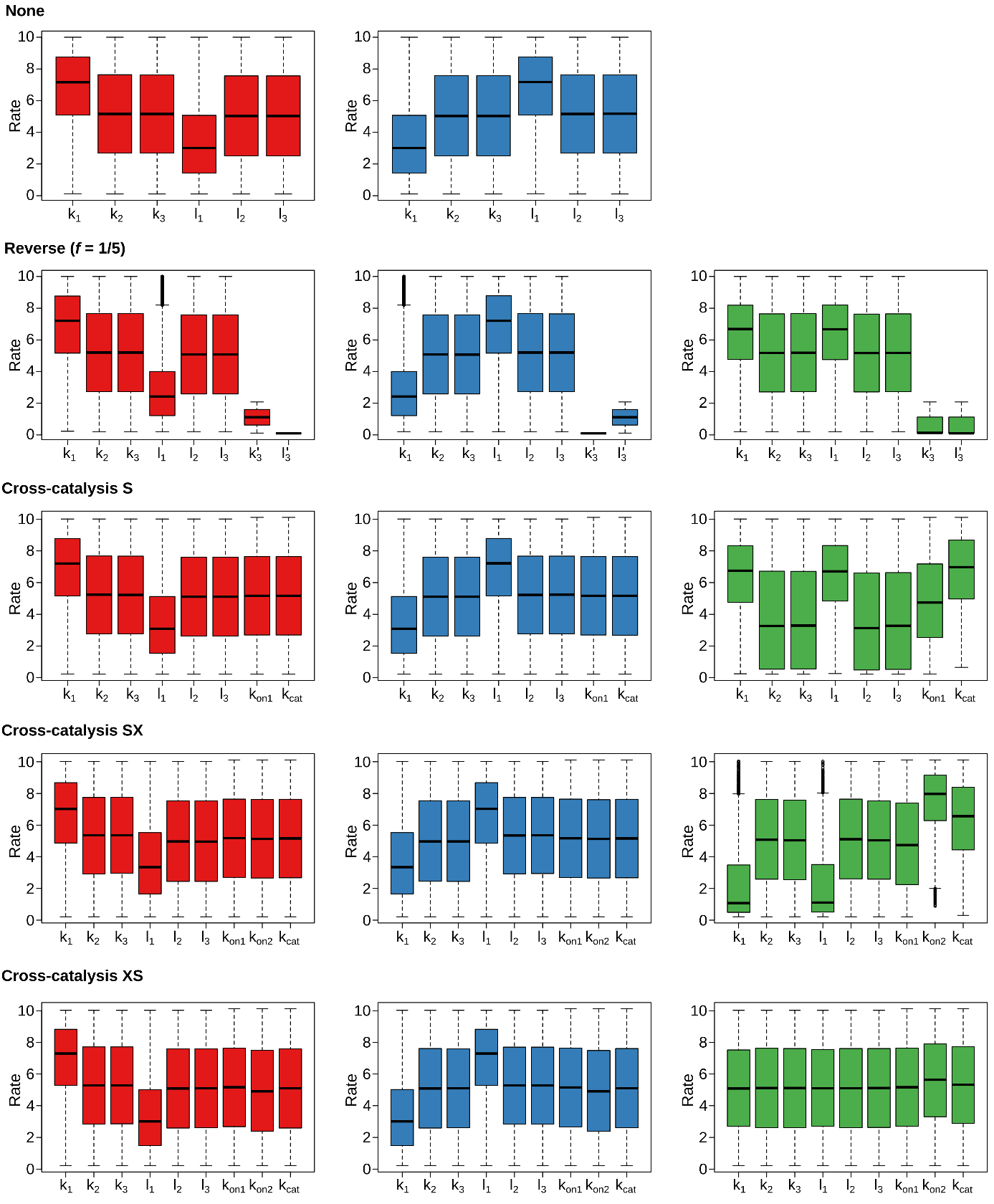


**Figure S7**. Patterns of kinetic rates in the investigated scenarios. Each row shows one scenario, and each column shows the box-plots of the distributions of kinetic rate values for the possible outcomes: cycle A wins (red), cycle B wins (blue) and cycle A and B coexist (green). The sizes of the cycles were the same: $n=m=3$.

**References**

Gardner, D.J., Reynolds, D.R., Woodward, C.S., Balos, C.J., 2022. Enabling new flexibility in the SUNDIALS suite of nonlinear and differential/algebraic equation solvers. ACM Transactions on Mathematical Software (TOMS). https://doi.org/10.1145/3539801

Schuster, P, 2019. What is special about autocatalysis? Chemical Monthly 150, 763–775. https://doi.org/10.1007/s00706-019-02437-z
